# Dual metal nanoparticles within multicompartmentalized mesoporous organosilicas for efficient sequential hydrogenation

**DOI:** 10.1038/s41467-021-25226-x

**Published:** 2021-08-17

**Authors:** Houbing Zou, Jinyu Dai, Jinquan Suo, Rammile Ettelaie, Yuan Li, Nan Xue, Runwei Wang, Hengquan Yang

**Affiliations:** 1 School of Chemistry and Chemical Engineering, Shanxi University, Taiyuan, China; 2 State Key Laboratory of Inorganic Synthesis and Preparative Chemistry, College of Chemistry, Jilin University, Changchun, China; 3 Food Colloids Group, School of Food Science and Nutrition, University of Leeds, Leeds, UK

**Keywords:** Catalyst synthesis, Heterogeneous catalysis, Porous materials, Nanoparticles

## Abstract

Controlling localization of multiple metal nanoparticles on a single support is at the cutting edge of designing cascade catalysts, but is still a scientific and technological challenge because of the lack of nanostructured materials that can not only host metal nanoparticles in different sub-compartments but also enable efficient molecular transport between different metals. Herein we report a multicompartmentalized mesoporous organosilica with spatially separated sub-compartments that are connected by short nanochannels. Such a unique structure allows co-localization of Ru and Pd nanoparticles in a nanoscale proximal fashion. The so designed cascade catalyst exhibits an order of magnitude activity enhancement in the sequential hydrogenation of nitroarenes to cyclohexylamines compared with its mono/bi-metallic counterparts. Crucially, an interesting phenomenon of neighboring metal-assisted hydrogenation via hydrogen spillover is observed, contributing to the significant enhancement in catalytic efficiency. The multicompartmentalized architectures along with the revealed mechanism of accelerated hydrogenation provide vast opportunity for designing efficient cascade catalysts.

## Abstract (web summary)

Controlling localization of multiple metal nanoparticles on a single support is at the cutting edge of designing innovatory cascade catalysts. Here, the authors report a multicompartmentalized mesoporous organosilica to spatially position different metal nanoparticles in intimate proximity for efficient sequential hydrogenation reactions.

## Introduction

Supported metal catalysts are at the heart of heterogeneous catalysis^1–3^. Spatially separated localization of multiple metal nanoparticles (NPs) on a single support is currently at the cutting edge of designing supported metal catalysts because such a kind of catalysts enables effective cascade of multistep reactions^4–9^. Their noteworthy advantages are shortening the diffusion distance of reaction intermediates and achieving synergistic catalysis between different metal NPs, both of which lead to boosted reaction efficiency and tunable selectivity^10–19^. An ideal supported multimetal cascade catalyst should exhibit the following features: (i) being capable of spatially isolating different metal NPs in intimate proximity; (ii) having short nanochannels to connect different metal NPs and thus guaranteeing efficient molecular transport between them; and (iii) enabling positive synergistic catalysis.

In pursuit of the ideal supported multimetal cascade catalysts, researchers have explored various nanostructured materials which are essential for achieving spatial localization of multiple metal NPs. For example, core-shell nanostructures were employed to spatially isolate two metal NPs within the core or on the outer shell for CO_2_ hydrogenation^20–25^. Similarly, TiO_2_ nanotubes were reported to localize Pt and Ni NPs on their outer or inner surfaces using atomic layer deposition for transfer hydrogenation^26–28^. Hierarchical porous silicas were also used to position dual metal NPs through stepwise modification of their pore surfaces for sequential oxidation of cinnamyl alcohol^29,30^. Despite the great success in constructing cascade catalysts via such spatial separation of multiple metal NPs, these existing materials either lack compartmentalized structures to accommodate metal NPs on the outer surfaces^20–28^, or else are unable to provide well-defined nanochannels for connecting different metal NPs in nanoscale proximity^26–30^. The unsatisfactory integration of all the desired features in a single catalyst hinders realization of efficient synergistic catalysis. Therefore, there is still a big remaining gap between these reported catalysts and the ideal cascade catalysts. In this context, developing new nanostructured materials to separately position multiple metal NPs in nanoscale proximity, so as to realize the full potential of cascade catalysts, is a rather challenging task.

In this work, we developed a novel multicompartmentalized mesoporous organosilica (MCMOS) to spatially isolate different metal NPs in intimate proximity for constructing efficient cascade catalysts. Possessing grooves on the outer surfaces and nanocavities within the interior, this material allowed us to position Ru and Pd NPs separately in the grooves and in the nanocavities, which are connected via nanopores on distance scales as short as 10 nm. The developed catalyst showed a remarkable catalytic efficiency in sequential hydrogenation of nitroarenes to cyclohexylamines, which otherwise was not achievable by its better known mono/bi-metallic counterparts. An interesting phenomenon involving neighboring metal-assisted hydrogenation was observed, in which hydrogen spillover from a catalytically inactive metal to the neighboring catalytically active one in nanoscale proximity accelerates the hydrogenation rate of each step of the cascade reaction. This mechanism was identified to be crucial for the enhanced catalytic efficiency.

## Results

### Preparation and characterization of MCMOS

**Fig. 1:**
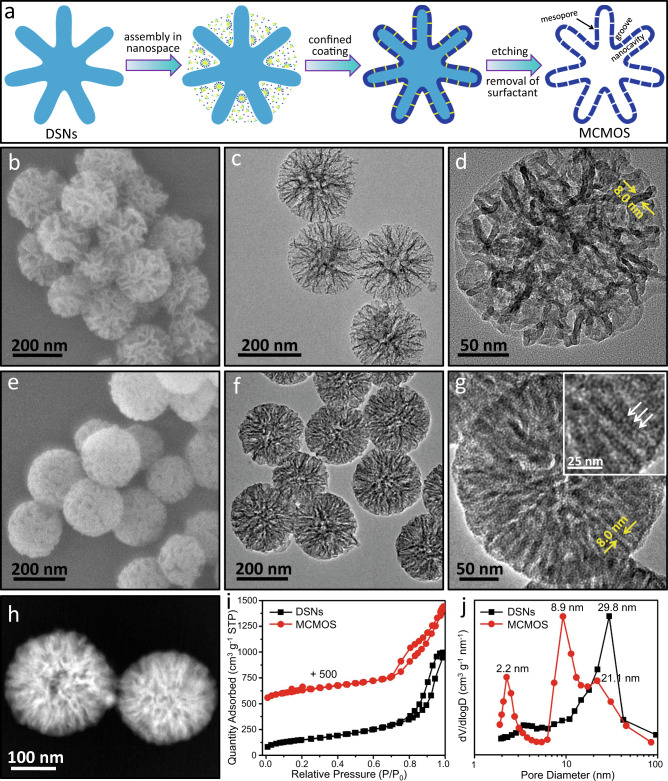
Synthesis and characterization of MCMOS. **a** Schematic illustration for the fabrication of MCMOS. **b**–**d** SEM (**b**) and TEM (**c**, **d**) images of DSNs. **e**–**h** SEM (**e**), TEM (**f**, **g**) and HAADF-STEM (**h**) images of MCMOS. **i** Nitrogen adsorption-desorption isotherm of DSNs and MCMOS. **j** BJH pore size distributions of DSNs and MCMOS. The inset in (**g**) is the HRTEM image showing nanochannels in the MOS layer.

To achieve a multicompartmentalized structure, we developed a confined coating-etching strategy to synthesize MCMOS, as illustrated in Fig. 1a. Dendritic mesoporous silica nanoparticles (DSNs) with pore diameter of 30 nm (Fig. 1b–d) were chosen as the hard template because of their large-sized dendritic channels^31^. Such channels allowed a sol-gel process to occur within the pores, hence generating a layer of mesoporous organosilica (MOS) on the channel surfaces by using organosilane 1, 2-bis(triethoxysilyl)ethane (BTEE) as the precursor. Due to the high pH conditions (pH≈14), the process of MOS growth simultaneously induced dissolution of the enveloped frameworks of DSNs which were hydrothermally less stable than MOS^32–34^. Such simultaneous growth of MOS and dissolution of DSNs yielded nanocavities within the formed MOS framework. However, owing to the sufficiently large pores of DSNs, the growth of MOS layer did not completely cram the initial channels of DSNs, leading to the formation of many grooves on the surfaces of MCMOS. In parallel, mesopores were generated in the formed MOS layer because of the introduction of hexadecyltrimethylammonium bromide (CTAB, acting as pore-directing agents), which served as nanochannels for connecting the interior nanocavities and the surface grooves with each other.

An examination of the SEM image of the synthesized MCMOS reveals that it is composed of uniform spherical particles with particle size of 250 ± 20 nm (Fig. 1e). A close inspection shows a number of grooves with an average size of ca. 20 nm, located on the surfaces of MCMOS. Comparing MCMOS with the original DSNs template, the size of formed grooves is much smaller than the channel size of DSNs. This is the result of the growth of MOS layer, taking place on the surface of channels inside DSNs. Interestingly, nanocavities are also clearly observed within the interior of MCMOS particles (Fig. 1f). The HRTEM image in Fig. 1g further reveals that the width of the formed nanocavities is almost equal to the thickness of the pore walls of DSNs. This result is an indication that the nanocavities stem from the dissolution of silica frameworks, occurring at the same time as the MOS growth. To the best of our knowledge, such a unique nanostructure, quite distinct from the previously existing core-shell/hollow structures^32–35^, has never been reported. Moreover, on the MOS framework, numerous mesopore channels connecting the interior nanocavities with the outer surface grooves, having a pore size of 2–3 nm and pore length of about 10 nm, are discernible (the inset in Fig. 1g). Such an architecture is further confirmed by the HADDF-STEM image (Fig. 1h). The sorption isotherm of MCMOS exhibits a typical type-IV curve with a hysteresis loop in the P/P_0_ range of 0.7–1.0. The Barrett–Joyner–Halenda (BJH) pore size distribution curve displays trimodal pore sizes centered at 2.2, 8.9, and 21.1 nm, which can be attributed correspondingly to the mesopores on MOS framework, the nanocavities within MCMOS, and the grooves on the MCMOS surfaces. Elemental mapping shows homogeneous distributions of Si, O, and C elements throughout the particle, indicating the framework is composed of organosilica (Supplementary Fig. 1). The organosilica framework is also verified collectively by separate results of Fourier transform infrared (FT-IR) spectroscopy, the solid-state NMR spectroscopy as well as the thermogravimetry analysis (Supplementary Fig. 2).

To identify key factors for the formation of such a unique architecture, we conducted a series of controlled experiments. By decreasing the amount of BTEE from 0.15 to 0.10 mL but keeping the other conditions unchanged, we found that the obtained MCMOS still exhibited a similar trimodal porous structure. However, in this case it was noted that the interior was sparse and the framework walls of MOS were thin (Supplementary Fig. 3a–c). Conversely, when the amount of BTEE was increased from 0.15 to 0.2 mL, a core-shell structure with a denser interior and a very thin MOS shell around the whole particle surface was generated (Supplementary Fig. 3d–f). This is because the excessive organosilane caused the coating process to continue on the external surfaces of DSNs, even after cramming the channels in their interior. Furthermore, we found that the channel size of DSNs also played a crucial role in the formation of MCMOS (Supplementary Fig. 4). MCMOS was successfully obtained using DSNs with a channel size of ≥20 nm as the template, but small channels (<15 nm) resulted in hollow nanospheres, as already reported in the literatures^32–35^. It is likely that the small channels failed to provide sufficiently large spaces for the co-assembly of surfactant and organosilica oligomers. Therefore, the coating-etching process occurred predominantly on the external surfaces of DSNs instead. Additionally, the framework of MCMOS materials can be diversified through altering organosilane precursors (Supplementary Figs. 5–6). Using bis(triethoxysilyl)methane (BTEM) or 1, 2-bis(triethoxysilyl)ethylene (BTEEE) as precursors was found to be viable for the synthesis of MCMOS. Multicompartmentalized mesoporous silica with pure inorganic framework was prepared by calcining the MCMOS materials in air at 550 °C for 6 h (Supplementary Fig. 7). MCMOS materials with larger nanochannels (pore diameter ~3.2 nm) connecting the surface grooves and the interior nanocavities could also be obtained using octadecyltrimethylammonium chloride (OTAC) with longer hydrophobic chains as the surfactant (Supplementary Fig. 8). These results suggest that the dosage of organosilane and the channel size of DSNs are crucial for the formation of multicompartmentalized architecture and that the structure of MCMOS materials is tunable.

### Preparation and characterization of the cascade catalyst

Our synthesis protocol, in combination with the unique architecture of MCMOS, allows us to stepwise position different metal NPs in the surface grooves and within the interior nanocavities, separately. Here, Ru and Pd were selected to demonstrate the construction of a cascade catalyst (Ru/Pd/MCMOS) since Ru NPs are proven to be catalytically active towards hydrogenation of arenes^36–39^ while Pd NPs are ideal catalysts towards hydrogenation of nitro group^40–42^. Integration of these two metal NPs in a single particle is necessary to realize efficient one-pot sequential hydrogenation of nitrobenzene to cyclohexylamine^43^.

**Fig. 2:**
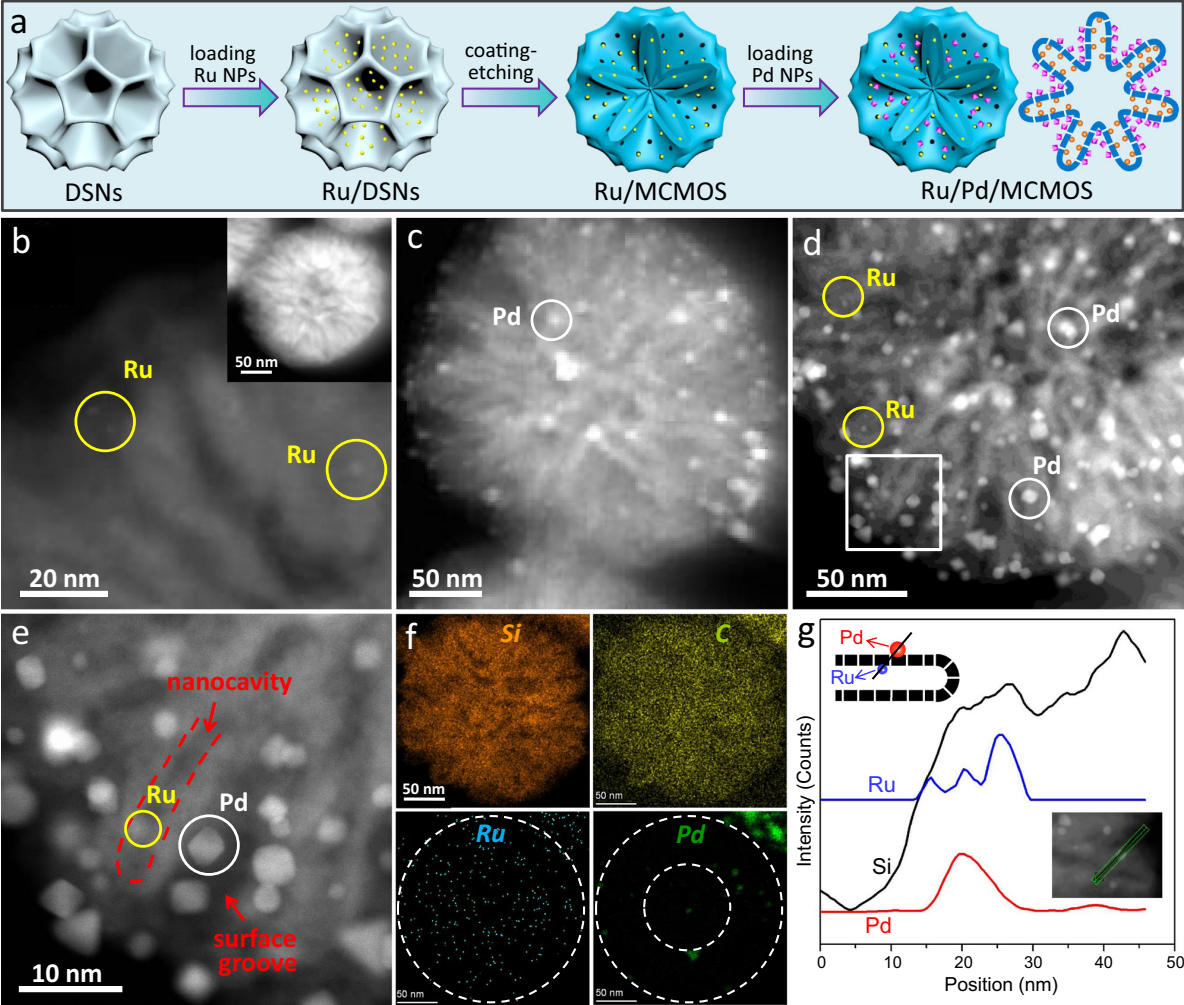
Synthesis and characterization of the Ru/Pd/MCMOS cascade catalyst. **a** Schematic illustration for the fabrication of Ru/Pd/MCMOS. **b**–**d** HAADF-STEM images of Ru/MCMOS (**b**), Pd/MCMOS (**c**) and Ru/Pd/MCMOS (**d**). **e**–**g** High-magnification HAADF-STEM image (**e**), EDX element mapping (**f**) and EDX line-scanning profile (**g**) of Ru/Pd/MCMOS.

As displayed in Fig. 2a, Ru NPs (≤2 nm) were first introduced into the nanocavities of MCMOS (Ru/MCMOS) by using as-prepared Ru-loaded DSNs (Ru/DSNs) as the template. To firmly anchor Ru NPs, aminopropyl group was grafted on the surface of the channels of DSNs before growing MOS and then was functionalized on the surface of MCMOS after the process of coating-etching (Supplementary Fig. 9). The desirable encapsulation effect of nanocavities and the coordination interaction of surface aminopropyl groups served to prevent the Ru NPs from leaching from the surface^44^. Subsequently, Pd NPs with particle sizes of 4–10 nm were loaded into the surface grooves via an adsorption process (see the Methods). Because Pd NPs in size were larger than the nanochannels on the MOS layer (2–3 nm), they had no possibility of entering the nanocavities located within the interior of MCMOS. Therefore, Pd NPs were solely confined inside the surface grooves. As a result, Ru and Pd NPs were spatially separated by the MOS layer, as depicted in Fig. 2a. The TEM images of such a fabricated Ru/Pd/MCMOS show that both Ru and Pd NPs are co-localized within a single particle (Supplementary Fig. 10). The spatially separated locations of these two metals within Ru/Pd/MCMOS are verified by the positional comparisons with their corresponding monometallic samples, Ru/MCMOS (Fig. 2b and Supplementary Fig. 11) and Pd/MCMOS (Fig. 2c and Supplementary Fig. 12). From the HADDF-STEM images (Fig. 2d, e), it is clearly seen that Ru NPs are confined within the nanocavities, whereas the Pd NPs with a stronger contrast are positioned in the grooves. This observation can be further supported by the X-ray photoelectron spectroscopy (XPS) results for Ru/Pd/MCMOS. Typical Pd 3*d* peaks with a binding energy of 336.1 eV for Pd 3*d*_5/2_ appeared in the XPS spectra of Ru/Pd/MCMOS and Pd/MCMOS while no obvious Ru 3*p* peaks were observed over Ru/Pd/MCMOS and Ru/MCMOS (Supplementary Fig. 13). This is due to the encapsulation of Ru NPs within the interior nanocavities. These results confirm that Ru NPs were confined in the interior of Ru/Pd/MCMOS while Pd NPs resided on its surface grooves, as expected. Particle size distributions for the Ru NPs and the Pd NPs of Ru/Pd/MCPMO reveal a bimodal distribution, with well-defined maxima at 1.5 and 6.2 nm, superimposable with those observed for monometallic Ru or Pd analogues (Supplementary Fig. 14). Furthermore, EDX elemental mapping displays that the Ru, Si and C elements are distributed throughout the Ru/Pd/MCMOS particle while the Pd element is predominantly visible in the outer half of the particle (Fig. 2f). This is because the grooves do not extend to the center of the particle when formed via a confined coating-etching process. The EDX line-scanning profile unveils that the distribution of Pd element is segregated away from Ru element (Fig. 2g) because they are kept apart by the MOS layer. Nonetheless, the two metals are still in close proximity, within nanoscale distances of each other, but Ru and Pd NPs are well separated from each other (inside and outside the nanocavities of MCMOSs connected by nanochannel).

### Enhanced catalytic efficiency

**Fig. 3:**
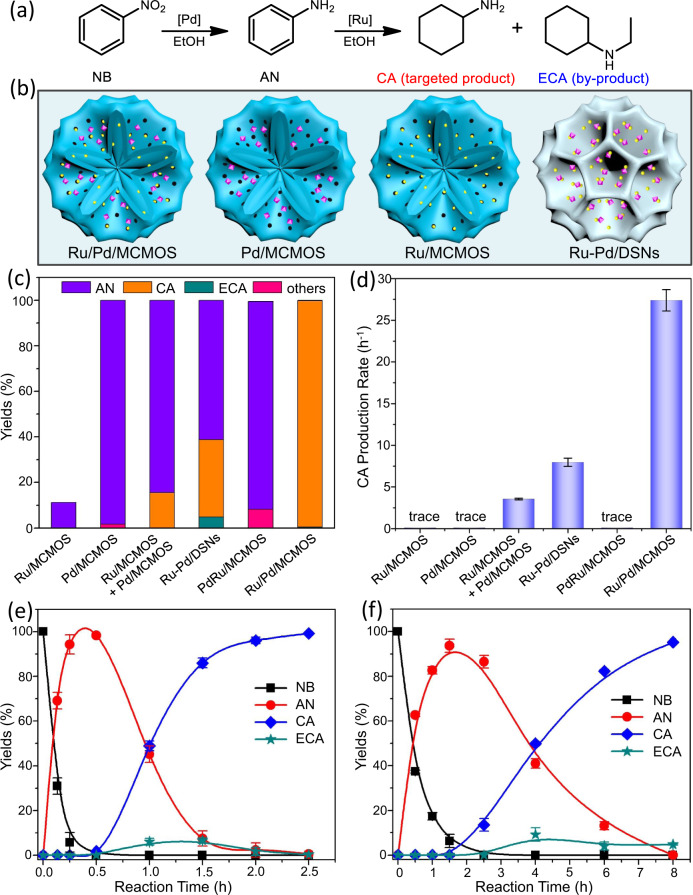
Catalytic performances evaluation. **a** Reaction network of the sequential hydrogenation of nitrobenzene. **b** Schematic illustration of four catalysts including Ru/Pd/MCMOS, Pd/MCMOS, Ru/MCMOS and Ru-Pd/DSNs. **c** Product distributions of the sequential hydrogenation over different catalysts. **d** The CA production rate over different catalysts. The production rate value is estimated according to the CA yield of 20–30%. **e** Kinetic plots of the sequential hydrogenation over Ru/Pd/MCMOS. **f** Kinetic plots of the sequential hydrogenation over the physically mixed catalyst. Reaction conditions: NB (0.25 mmol), solid catalyst (Ru 0.77 mol%, Pd 1.01 mol%), ethanol (2.0 mL), H_2_ (2.0 MPa), 80 °C, 2.5 h.

Sequential hydrogenation of nitroaromatic compounds is an important transformation for producing cycloaliphatic primary amines, necessary intermediates accessible to various high-value chemicals^45^. Such a transformation consists of two steps of hydrogenation^46–49^, i.e. nitro hydrogenation and aromatic ring hydrogenation (Fig. 3a). Dual metal-involved catalysts are necessary to achieve high catalytic efficiency^43^, because they can respectively catalyze one step of the sequential hydrogenation aforementioned.

Our Ru/Pd/MCMOS cascade catalyst enabled the sequential hydrogenation of nitrobenzene (NB) to proceed smoothly under mild reaction conditions (80 °C, 2.0 MPa), giving the final product cyclohexylamine (CA) in 99.2% yield along with trace aniline (AN, intermediate) and ethylcyclohexylamine (ECA, side product) within 2.5 h reaction period (Fig. 3c and Supplementary Table 1). The CA production rate (defined here as the mole number of generated CA per hour) was calculated to be as high as 27.4 h^−1^ (Fig. 3d). For benchmarking the performance of our Ru/Pd/MCMOS, we also examined a set of catalysts including monometallic and bimetallic catalysts (Fig. 3b). Under the same reaction conditions, the monometallic Pd/MCMOS catalyst gave intermediate aniline (AN) in ≥99% yield, and only a trace amount of the final product CA was detected (Fig. 3c). Another monometallic Ru/MCMOS catalyst provided a NB conversion of only 11.2% and no product CA could be detected in this case (Fig. 3c). This is reasonable because Pd/MCMOS was only active for converting NB to AN (the first step) while Ru/MCMOS was exclusively active in the AN hydrogenation (the second step, Supplementary Fig. 15), which is consistent with previous literatures^37–42^. Over the physically mixed catalyst (a mixture of Pd/MCMOS and Ru/MCMOS), a yield of 15.5% for the final product CA was obtained within the same reaction time although the conversion in the first step reached 99.9% (Fig. 3c). The CA production rate for the physically mixed catalyst was only 3.55 h^−1^, which was 7.72-fold lower than that of our Ru/Pd/MCMOS (Fig. 3d). Moreover, we tested another two bimetallic catalysts (their preparation procedures are provided in Supplementary Experimental Section): Ru-Pd/DSNs catalyst with Ru and Pd NPs jointly loaded inside the same channels of DSNs (without spatial isolation as shown in Supplementary Fig. 16) and PdRu/MCMOS alloy catalyst (Supplementary Fig. 17). Ru-Pd/DSNs gave a CA yield of 34.0% with a production rate of 7.95 h^−1^, and PdRu/MCMOS even showed almost no activity for producing CA (Fig. 3c, d). Obviously, despite containing the dual metals, their activities for this sequential hydrogenation were much lower than our cascade catalyst. This can be explained in terms of electronic effects. As shown by TEM images of Ru-Pd/DSNs (Supplementary Fig. 16), the small Ru and large Pd NPs are so close in this non-compartmentalized sample, as to be in direct contact with each other. This situation induces charge transfer between these two metals, as evidenced by the XPS results (Supplementary Fig. 18). As a result, the surface electron density of Ru NPs decreases, leading to inability in the adsorption and activation of aromatic ring^50,51^ (Supplementary Fig. 19). It is the same case with the PdRu/MCMOS alloy catalysts^52^. These comparisons highlight the phenomenon that the presence of the second catalytically inert metal, positioned in nanoscale proximity but yet not in direct contact with the first metal, can significantly enhance the overall catalytic efficiency.

This phenomenon was also observed in the hydrogenation of other nitroarenes. Nitroarenes with different substituted groups including methyl, ethyl and even dimethyl could all be efficiently converted into their corresponding cyclohexylamines with yields of ≥93% over our cascade catalyst (Table 1, Entries 1–6). For the challenging substrates such as *p*-nitrophenol and *p*-methoxynitrobenzene that are easy to deoxidize by hydrogenolysis under high-temperature, our catalyst still gave cyclohexylamines in selectivities as high as ≥98.1% (Entries 7, 8). Alicyclic diamines were also obtained in an approximately quantitative yield from different nitroanilines (Table 1, Entries 9, 10). Using 2, 6-di-tert-butyl–4-nitrophenol with a large molecule size as substrate, the Ru/Pd/MCMOS catalyst also presented a yield of 99.4% for corresponding cyclohexylamine within 12.0 h (Table 1, Entry 11). In contrast, for all these investigated substrates, the physically mixed catalyst only gave yields of 0.5–29.1%, which were 3.4–193 fold lower than those obtained over Ru/Pd/MCPMO. Collectively, these results point to the general fact that our cascade catalyst has an enhanced catalytic efficiency in comparison to the physically mixed catalyst. Moreover, the Ru/Pd/MCPMO cascade catalyst showed a good recyclability. The CA yield obtained within 1.5 h was slightly decreased from initial 84.4% to 74.0% after six successive reaction cycles (Supplementary Fig. 20a). Such a high catalytic stability was further confirmed by the filtration test (Supplementary Fig. 20b). By analyzing the residual filtrate of the sixth reaction cycle using ICP-MS, only ppb Pd and Ru species could be detected. These results indicated that only 0.5 wt% of metal NPs were leached out from the catalyst. The TEM images and XPS spectra in Supplementary Fig. 21 reveal that the recovered catalyst remains a well-defined multicompartmentalized structure and most of the Ru and Pd NPs are still confined in their own sub-compartments. Ru NPs are observed to undergo slight sintering from the high-magnification TEM image. Accordingly, the Ru/Pd/MCMOS cascade catalyst is catalytically stable and recyclable, and the slight activity loss after the sixth reaction cycle should be attributed to the slight metal leaching and sintering.

**Table 1:** Results of sequential hydrogenation of various nitroarenes over the Ru/Pd/MCMOS cascade catalyst and the physically mixed catalyst^a^.

Reaction conditions: nitroarenes (0.25 mmol), solid catalyst (Ru 0.77 mol%, Pd 1.01 mol%), ethanol (2.0 mL), H_2_ (2.0 MPa), 80 °C.

^a^Numbers in brackets refer to the results obtained over the physically mixed catalyst.

^b^Determined by GC and GC–MS.

### Neighboring metal-assisted hydrogenation via hydrogen spillover

The significantly enhanced catalytic efficiency of our Ru/Pd/MCMOS, in comparison to the physically mixed catalyst, promotes one to clarify the origins of such a pronounced improvement. We checked the kinetics of sequential hydrogenation over Ru/Pd/MCMOS and the physically mixed catalyst. In both these two systems, the maximum concentrations of intermediate AN reached approximately 100% during the reaction process (Fig. 3e, f), meaning that any significant AN hydrogenation only occurred once the NB hydrogenation had been almost completed. This tends to indicate that there existed a substrate inhibition effect for the second step. Our poisoning experiment provides additional support for the occurrence of this substrate inhibition effect. As shown in Supplementary Figs. 22–24, the AN hydrogenation is completely poisoned in the presence of 50% of *p*-nitrotoluene. These results exclude the possibility that timely transport of intermediate from the Pd NPs to the Ru NPs may result in the enhanced catalytic efficiency. It is well recognized that the concentration of active hydrogen species on the metal surfaces is crucial to hydrogenation^53–55^. Based on the enhanced activity in the presence of the catalytically inert second metal as discussed above, we speculate that the migration of active hydrogen from neighboring metal NPs may substantially accelerate the reactions of both of steps and thereby improve the overall reaction rate.

**Fig. 4:**
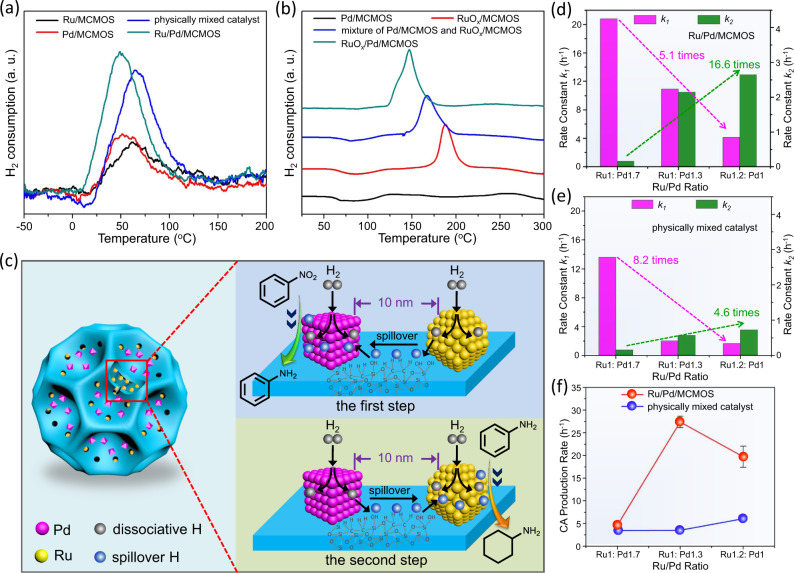
Neighboring metal-assisted hydrogenation via hydrogen spillover. **a** H_2_-TPD profiles of Pd/MCMOS, Ru/MCMOS, the physically mixed catalyst (Pd/MCMOS + Ru/MCMOS) and Ru/Pd/MCMOS. **b** H_2_-TPR profiles of Pd/MCMOS, RuO_x_/MCMOS, the mixture of Pd/MCMOS and RuO_x_/MCMOS, and RuO_x_/Pd/MCMOS. **c** Schematic illustration for neighboring metal-assisted hydrogenation over Ru/Pd/MCMOS. **d** The rate constant of NB (*k*_*1*_) and AN transformation (*k*_*2*_) in the sequential hydrogenation reaction over Ru/Pd/MCMOS with different Ru/Pd ratios. **e** The rate constant of NB (*k*_*1*_) and AN transformation (*k*_*2*_) in the sequential hydrogenation reaction over the physically mixed catalysts with different Ru/Pd ratios. **f** The relationship between the Ru/Pd ratio and the CA production rate in the sequential hydrogenation reaction.

To validate our hypothesis, hydrogen spillover between the two metal NPs was investigated by hydrogen temperature-programmed desorption (H_2_-TPD) and temperature-programmed reduction (H_2_-TPR), which have been shown to be helpful in investigating the hydrogen spillover effect^56,57^. As displayed in Fig. 4a, Pd/MCMOS and Ru/MCMOS exhibit a desorption peak centered at 51 °C and 61 °C, respectively. This indicates that both of metal NPs have good ability for H_2_ activation and dissociation, which can be further evidenced by the color change in the mixture of the catalysts and WO_3_ nanoparticles (Supplementary Fig. 25) and the theoretical calculations (Supplementary Fig. 26). Interestingly, Ru/Pd/MCMOS shows a desorption peak centered at 49 °C, which is lower than that of the physically mixed catalyst (63 °C). This observation suggests that the ability in H_2_ adsorption and dissociation is improved when positioning Ru and Pd NPs in close nanoscale proximity. To further confirm the hydrogen spillover occurring between these two metal NPs, the Ru NPs were partly oxidized to RuO_x_ species (as evidenced by the XPS spectrum in Supplementary Fig. 27) by hypochlorous acid for H_2_-TPR tests. It can be observed that there is a distinct peak centered at 187 °C in the H_2_-TPR profile of RuO_x_/MCMOS (Fig. 4b), which can be attributed to the reduction of RuO_x_ species^58^. After introducing Pd NPs in the nanoscale proximity to RuO_x_, the temperature needed for the RuO_x_ reduction on RuO_x_/Pd/MCMOS is considerably lowered down to 147 °C. This again confirms the hydrogen spillover occurring from Pd to RuO_x_. Notably, the reduction peak of RuO_x_/Pd/MCMOS is also lower than that for the systems involving a mixture of RuO_x_/MCMOS and Pd/MCMOS (167 °C), indicating that the closer proximity of two metals is more favorable to the hydrogen spillover.

This hydrogen spillover effect is also supported by the results of catalytic reactions. After Ru NPs were partly oxidized to RuO_x_ species, the conversion for AN hydrogenation over RuO_x_/MCMOS was dramatically decreased from 97% to 60.4% within a 2 h period (Supplementary Fig. 28). The decrease was due to the relatively poor H_2_ activation ability of RuO_x_ species. In contrast, a high conversion of 91.2% was obtained over RuO_x_/Pd/MCMOS, on which the Pd NPs were positioned beside the RuO_x_ species in close proximity. Since the Pd NPs themselves were inert for transforming AN to CA (as aforementioned), this activity enhancement must stem from the spillover of active hydrogen atoms generated on the surfaces of Pd NPs. These results consolidate our hypothesis that the migration of active hydrogen atoms, occurring between closely spaced but separately positioned neighboring metal NPs, can substantially accelerate hydrogenation reactions.

To further confirm the hydrogen spillover effect in Ru/Pd/MCMOS, we examined the reaction kinetics of the two catalytic steps independently over Ru/Pd/MCMOS and the physically mixed catalyst. As displayed in Supplementary Fig. 29, since all the reactions follow pseudo first-order kinetics, the reaction rate constants are determined to evaluate their catalytic efficiencies in accordance to the rate equation ln(C/C_0_) = *k*t. Our Ru/Pd/MCMOS catalyst shows a rate constant in the first step (*k*_*1*_), which is 3.77 times higher than that of the monometallic Pd/MCMOS catalyst. In the second step, the rate constant (*k*_*2*_) is also 2.42 fold larger than that of the monometallic Ru/MCMOS catalyst. Note that although the physically mixed catalyst also modifies both of the reaction rate constants (by 2.48 fold in the first step and 1.48 fold in the second step), the enhancements are much less pronounced than our Ru/Pd/MCMOS catalyst, which is due to its far distance between two metals in separate particles. These results clearly indicate that catalytically inert second metal in nanoscale proximity accelerates the hydrogenation reaction occurring on the neighboring metal NPs. Furthermore, we also found that the reaction rates of both catalytic steps over Ru/Pd/MCMOS were related to the ratios of Ru/Pd. For the first step of hydrogenating NB to AN, when keeping the Pd NPs loading unaltered while adjusting the catalytically inert Ru NPs loading from 0.3 wt% to 1.1 wt%, the rate constant was increased by 1.42-times (Supplementary Fig. 30). Likewise, for the second step of converting AN to CA, when keeping the Ru NPs loading constant, an increase of catalytically inert Pd NPs loading from 0.35 wt% to 1.01 wt% resulted in a 1.65 fold reaction rate enhancement (Supplementary Fig. 31). These findings further disclose that more the catalytically inert second metal is, higher the reaction rate of the hydrogenation occurring on neighboring metal NPs is, because higher concentration of active hydrogen atoms is provided by the second metal.

Based on the above results, we propose a mechanism of neighboring metal-assisted hydrogenation. As illustrated in Fig. 4c, the catalytically inert Ru NPs in the first step can still dissociate H_2_ efficiently, generating active hydrogen atoms that are subsequently migrated to the surfaces of Pd NPs located in close proximity. This leads to a high concentration of active hydrogen atoms on the Pd NPs during their catalytic reactions, thereby accelerating the hydrogenation of NB molecules adsorbed on the Pd surfaces. In the same manner, the catalytically inactive Pd NPs in the second step also activate H_2_ to promote the hydrogenation of AN molecules adsorbed on the Ru surfaces. Such a neighboring metal-assisted hydrogenation is strongly dependent on the distance and ratio between two metals because they are relevant to the generation and subsequent migration of active hydrogen atoms. This hydrogen spillover from the neighboring metal mainly contributed to the high overall catalytic efficiency obtained over Ru/Pd/MCMOS.

To examine the generality of this neighboring metal-assisted hydrogenation, we prepared the Ru/Au/MCMOS cascade catalyst by replacing the Pd NPs with Au NPs that were documented to have poor activity for hydrogen activation^59^. The TEM images indicate that both of the metal NPs were co-localized on the MCMOS support in a spatially isolated but nanoscale proximal fashion (Supplementary Fig. 32). Although both the Au NPs and the Ru NPs were catalytically inactive for nitrobenzene hydrogenation, as shown in Supplementary Fig. 33a, the Ru/Au/MCMOS catalyst exhibited a high activity for producing aniline from nitrobenzene. Furthermore, its activity was also much higher than that of the physical mixture of Ru/MCMOS and Au/MCMOS due to the closer proximity of two metal NPs of Ru/Au/MCMOS. More interestingly, the Ru/Au/MCMOS cascade catalyst even exhibited a reasonably good activity in the sequential hydrogenation of nitrobenzene for producing cyclohexylamine (Supplementary Fig. 33b). These results demonstrated the generality of the strategy of neighboring metal-assisted hydrogenation within multimetal cascade catalysts.

Furthermore, the proposed neighboring metal-assisted hydrogenation enabled us to tune the kinetics of this cascade reaction in a rather unconventional way. As shown in Supplementary Fig. 34, the reaction kinetics of NB sequential hydrogenation is very different when adjusting the Ru/Pd ratio of Ru/Pd/MCMOS but keeping the total metal loading constant. The rate constant of the first step (*k*_*1*_) is decreased from 20.8 to 4.11 h^−1^ when the ratio of Ru/Pd ratio is increased from 1/1.7 to 1.2/1 (Fig. 4d). At the same time, the rate constant of the second step (*k*_*2*_) is found to significantly increase from 0.16 to 2.65 h^−1^ (Fig. 4d). The decline of *k*_*1*_ and the rise of *k*_*2*_ in themselves are not surprising because the corresponding metals catalyzing each step are decreased or increased. However, the observations become far more interesting when they are compared to the corresponding results, involving the same change in the ratio of Ru/Pd in the physically mixed catalyst (Supplementary Fig. 35). As shown in Fig. 4e, the *k*_*1*_ value of the physically mixed catalyst declines much more dramatically than that of Ru/Pd/MCMOS (8.2 times vs 5.1 times), with its *k*_*2*_ value also rising far less (4.6 times vs 16.6 times). Consequently, for the overall cascade reaction, Ru/Pd/MCMOS exhibits a stronger dependence of CA production rate as a function of Ru/Pd ratio, in comparison to the physically mixed catalyst (Fig. 4f). This reflects the high sensitivity of our cascade catalyst to the variation in the metal ratios, thus providing an opportunity and a mean to optimize the kinetics of cascade reactions in a more effective way.

## Discussion

In summary, a novel multicompartmentalized nanoarchitecture MCMOS has been successfully synthesized to spatially position dual metal NPs for tandem reaction. MCMOS features grooves on the surfaces and nanocavities within its interiors, which are connected via uniform mesopores with pore lengths of about 10 nm. Such a unique architecture enables spatial isolation of different metals such as Ru and Pd within their sub-compartments. The so designed Ru/Pd/MCMOS cascade catalyst exhibits even an order of magnitude enhancement in catalytic efficiency in the sequential hydrogenation of nitroarenes to cyclohexylamines in comparison to the monometallic catalysts and other bimetallic counterparts. Importantly, we find a novel phenomenon of neighboring metal-assisted hydrogenation, which involves hydrogen spillover from a catalytically inactive metal to the neighboring catalytically active one for each step of the cascade reaction. Thus, even the metal, seemingly inert to catalyzing one of the two steps, begins to play an indirect synergic role in enhancing the reaction rate of the other step, leading to the significantly boosted catalytic efficiency. Our work not only demonstrates the design of a new architectured material for achieving the co-localization of multiple metal NPs but also supplies fresh insight into multimetal-involved tandem hydrogenation process, providing new opportunity for designing more efficient cascade catalysts.

## Methods

### Synthesis of MCMOS nanoparticles

DSNs were prepared according to a previous report^31^. In a typical synthesis of MCMOS, 100 mg of the as-prepared DSNs was homogeneously dispersed in a mixture containing 22 mL of water and 11 mL of ethanol by ultrasonication. After addition of 0.5 g of CTAB and 4.0 mL of ammonium aqueous solution (25–28%), the resultant mixture was further stirred at room temperature for 30 min. 0.15 mL of 1, 2-bis(triethoxysilyl)ethane (BTEE) was then added dropwise. After complete hydrolysis of BTEE, the obtained white suspension was transferred to a stainless steel autoclave with a Teflon container and was further hydrothermally treated at 100 °C for 24 h. The resultant MCMOS were collected through centrifugation and then washed with water and ethanol several times. The surfactant CTAB was removed by refluxing 0.5 g of the as-made material in 100 mL of ethanol containing 0.5 g of concentrated HCl aqueous solution for 6 h.

### Preparation of the Ru/Pd/MCMOS cascade catalyst

Firstly, Ru NPs were supported on the dendritic channel surfaces of DSNs via an impregnation-reduction method. To stably anchor Ru NPs, aminopropyl group was grafted on the pore surfaces of DSNs (NH_2_-DSNs). Typically, 0.5 mL of 3-aminopropyltriethoxysilane (APTES) was dropwise added into 20 mL of toluene solution containing 0.1 mL of triethylamine and 200 mg of DSNs, and the obtained mixture was refluxed for 6 h at 120 °C. Centrifugation was conducted to collect the NH_2_-DSNs sample. After that, a dosage of RuCl_3_ solution was added into 50 mL of aqueous solution containing 200 mg of NH_2_-DSNs, and the obtained mixture was stirred at 50 °C for 4 h. After the addition of NaBH_4_ solution (0.1 M, 2.0 mL), the mixture was further stirred for 24 h to reduce Ru ions, leading to Ru/DSNs. Then, the prepared Ru/DSNs were used as templates to prepare the Ru/MCMOS catalyst (Ru NPs-supported MCMOS) under the same conditions as MCMOS. Finally, 4–8 nm of Pd NPs suspension obtained according to a previous report^60^ was added drop-by-drop into a mixture containing 20 mL of H_2_O and a certain amount of Ru/MCMOS. The solid catalyst was collected by centrifugation and then washed with water and ethanol for several times. After drying at 80 °C for 6 h under vacuum, the Ru/Pd/MCMOS catalyst was obtained. The Pd/MCMOS catalyst (Pd NPs-supported MCMOS) was prepared under the same conditions as those for Ru/Pd/MCMOS except by replacing Ru/MCMOS with the MCMOS material. By inductively coupled plasma mass spectrometry (ICP-MS) analysis, the loading amount of Ru and Pd was identified to be 0.54 wt % and 0.75 wt% in these bimetallic/monometallic catalysts, respectively.

### Sequential hydrogenation of nitroarenes to cyclohexylamines

In a typical hydrogenation reaction, nitroaromatic (0.25 mmol), catalyst (36 mg, Ru 0.77 mol%, Pd 1.01 mol%) and ethanol (2.0 mL) were added to a Teflon-lined steel autoclave. Before reaction, the autoclave was sealed and flushed with H_2_ three times to remove the air. Then the autoclave was charged with a 2.0 MPa H_2_ at room temperature, and then heated from room temperature to 80 °C within 20 min and kept at this temperature under magnetic stirring (900 rpm). After the completion of the reaction, the autoclave was cooled down to room temperature. The products were analyzed by a gas chromatograph (Agilent 7890 A) equipped with an HP-5 column, and further confirmed by GC–MS (Agilent 7890 A GC/5973 MS). After each run the catalyst was separated from the reaction mixture by centrifugation, thoroughly washed with ethanol for five times, and dried at 60 °C under a vacuum for the next reaction cycle. After the sixth reaction cycle, the catalyst was recovered by centrifugation for characterization and the residual filtrate was allowed for further reaction under the same conditions. All of the error bars represent the standard deviations from three repeated experiments.

## Supplementary information

Supplementary Information

## Data Availability

The data that support the findings of this study are available from the corresponding author under reasonable request.
