# Supplementary material for: Dual metal nanoparticles within multicompartmentalized mesoporous organosilicas for efficient sequential hydrogenation
Source: Nat Commun. 2021 Aug 17;12:4968. doi: 10.1038/s41467-021-25226-x (PMC8371113; doi:10.1038/s41467-021-25226-x)
Supplement: Supplementary file 1 — Supplementary Information [file 41467_2021_25226_MOESM1_ESM.pdf]

Supplementary Information for

Dual metal nanoparticles within multicompartmentalized mesoporous organosilicas for efficient sequential hydrogenation

Houbing Zou[1], Jinyu Dai[2], Jinquan Suo[2], Rammile Ettelaie[3], Yuan Li[1], Nan Xue[1], Runwei Wang[2]*, and Hengquan Yang[1]*

[1] *School of Chemistry and Chemical Engineering, Shanxi University, Taiyuan, 030006, China*

[2] *State Key Laboratory of Inorganic Synthesisand Preparative Chemistry, College of Chemistry, Jilin University, Changchun, 130012, China.*

[3] *Food Colloids Group, School of Food Science and Nutrition, University of Leeds, Leeds LS2 9JT, United Kingdom.*

*To whom correspondence should be addressed: rwwang@jlu.edu.cn; hqyang@sxu.edu.cn

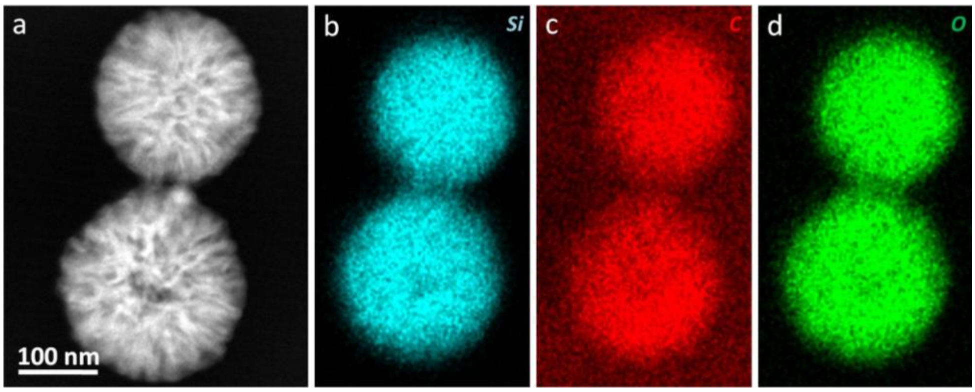

Supplementary Figure 1 | Characterization of MCMOS. (a) HAADF-STEM image and (b-d) EDX elemental mapping of MCMOS prepared using organosilane BTEE as the precursor.

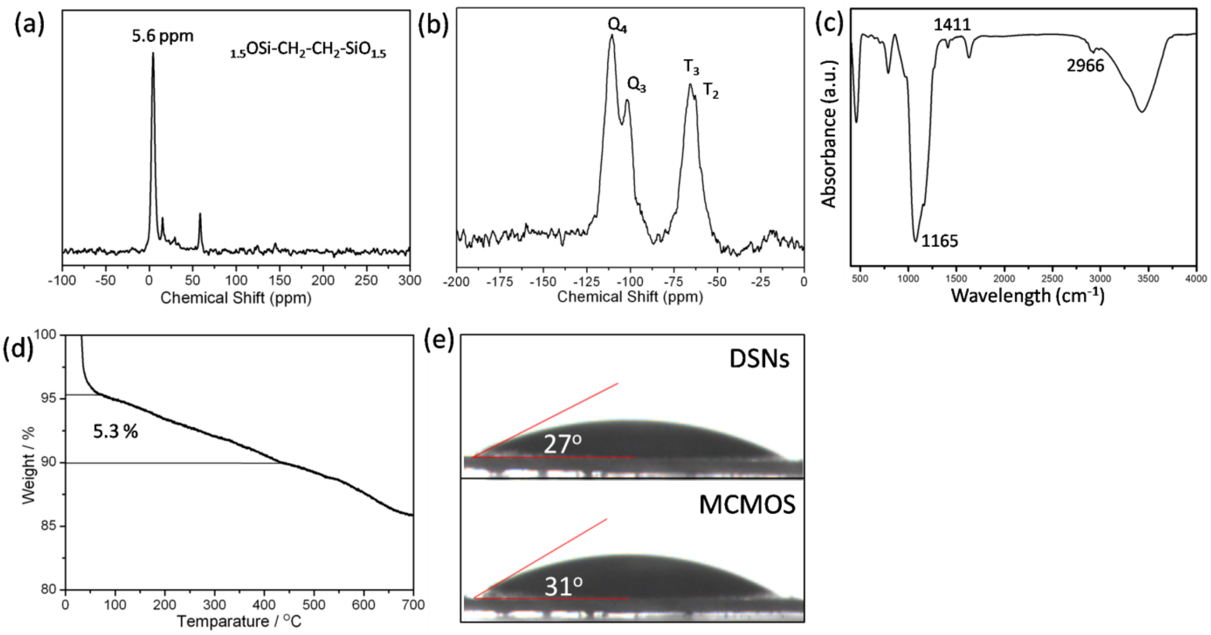

Supplementary Figure 2 | Characterization of MCMOS. (a) Solid state ^{13}C NMR spectrum, (b) solid state ^{29}Si NMR spectrum, (c) FT-IR spectrum and (d) TG curve of MCMOS prepared using organosilane BTEE as the precursor. (e) Water conctact angles of DSNs and MCMOS.

Noting: A distinct resonance appeared at 5.6 ppm in the ^{13}C NMR spectrum (Supplementary Fig. 2a), which could be attributed to the C species of the ethylene moiety. The FT-IR spectrum in Supplementary Fig. 2c showed several bands at 1410 cm^{-1}, 1160 cm^{-1} and 2966 cm^{-1}, which could be assigned to C–H vibration, Si–C vibration and C–H stretching vibration, respectively. These results proved the incorporation and integrity of the ethylene organic groups into the MOS framework. Additionally, the ^{29}Si NMR spectrum showed the existence of both T^n and Q^n sites in the range of -50 to -80 ppm and -90 to -125 ppm (Supplementary Fig. 2b), which we attributed to *Si*C(OSi)$_2$(OH) (T^2) and *Si*C(OSi)$_3$ (T^3) species of silicon attached to ethylene, and the (HO)*Si*(OSi)$_3$ (Q^3) and *Si*(OSi)$_4$ (Q^4) silicon species, respectively. The appearance of Q^n sites demonstrated that the dissolved silica species from DSNs co-condensed with hydrolyzed organosilane (BTEE) oligomers and then were transformed into the MOS framework. The TG curve showed a weight loss of only 5.3% from 100 to 450 ^{o}C, lower than the weight proportion of ethylene in the unit $O_{1.5}$Si–CH_2CH_2–$SiO_{1.5}$ (21%). This indicated the existence of SiO_2 units in the MOS framework. These results directly proved that the dissolved silica species have transformed into the MOS framework[1-3]. The very low content of ethylene (5.3%) was evidenced to show little impact on the hydrophobicity of the obtained MCMOS materials (Supplementary Fig. 2e).

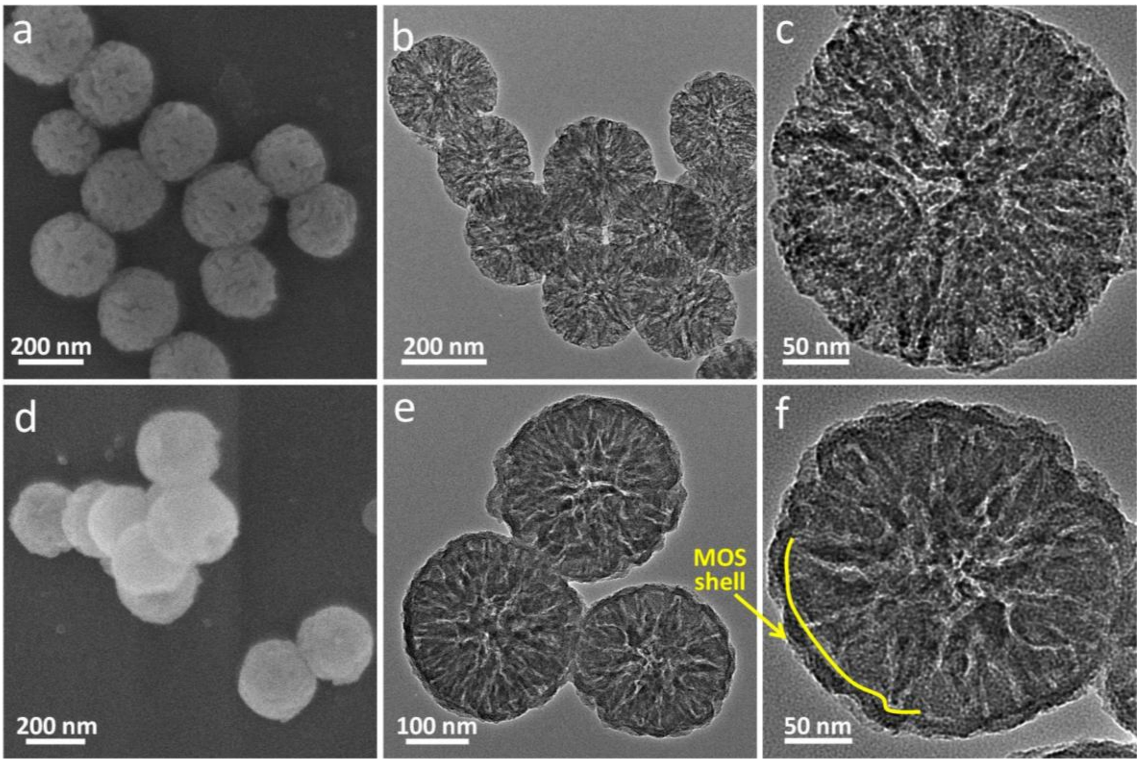

Supplementary Figure 3 | Characterization of MCMOS with different structural parameters. (a) SEM and (b, c) TEM images of the MCMOS materials prepared using 0.1 mL of organosilane BTEE under the standard conditions. (d) SEM and (e, f) TEM images of the MCMOS materials prepared using 0.2 mL of organosilane BTEE.

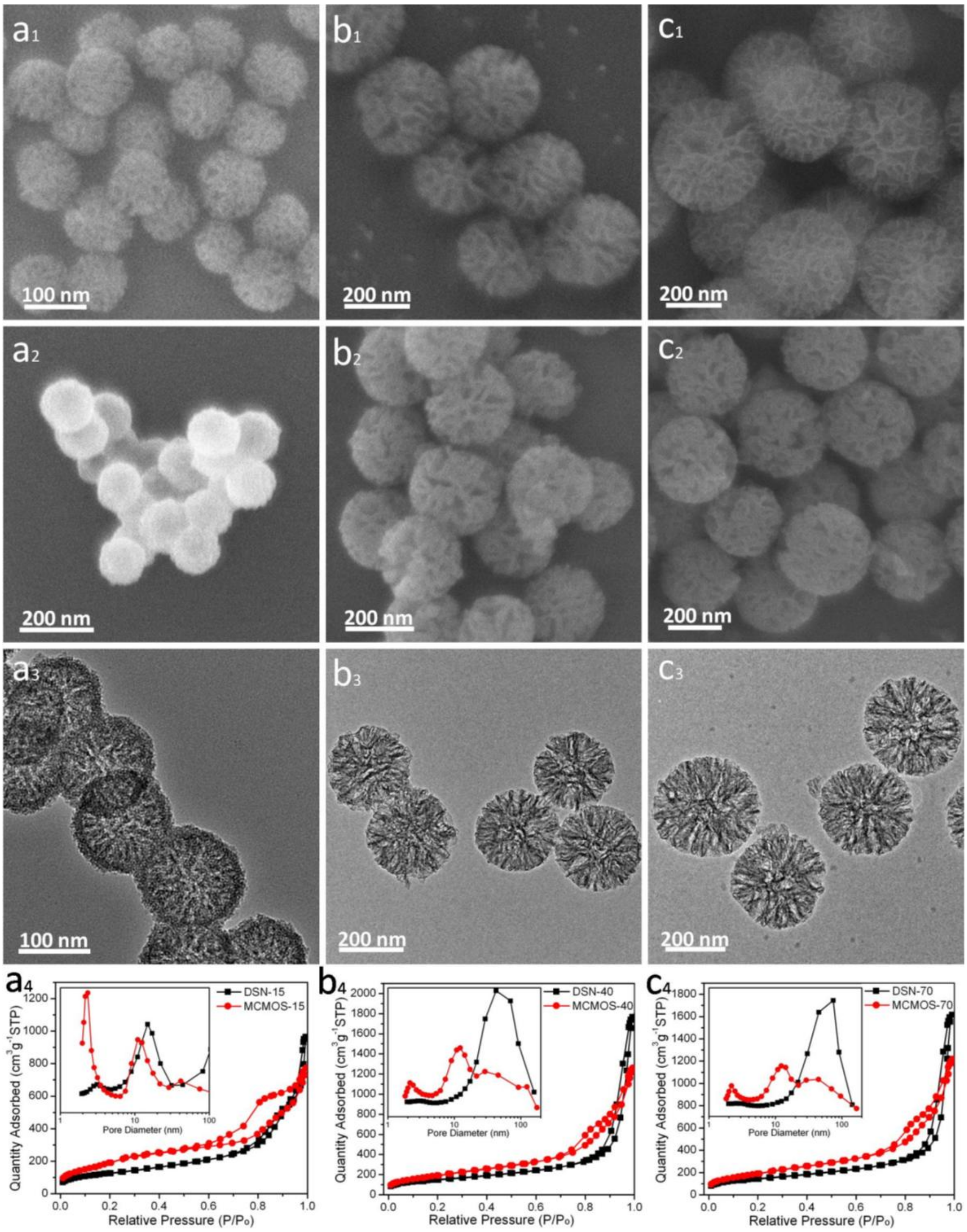

Supplementary Figure 4 | Characterization of MCMOS prepared under different conditions. (a_1, b_1, c_1) SEM images of DSNs with different channel sizes: 15 nm (a_1), 40 nm (b_1) and 70 nm (c_1). (a_2, b_2, c_2) SEM and (a_3, b_3, c_3) TEM images of the MCMOS materials prepared using DSNs with different channel sizes as the template. (a_4, b_4, c_4) Nitrogen adsorption-desorption isotherms and BJH pore size distribution curves of DSNs with different channel sizes and corresponding MCMOS materials.

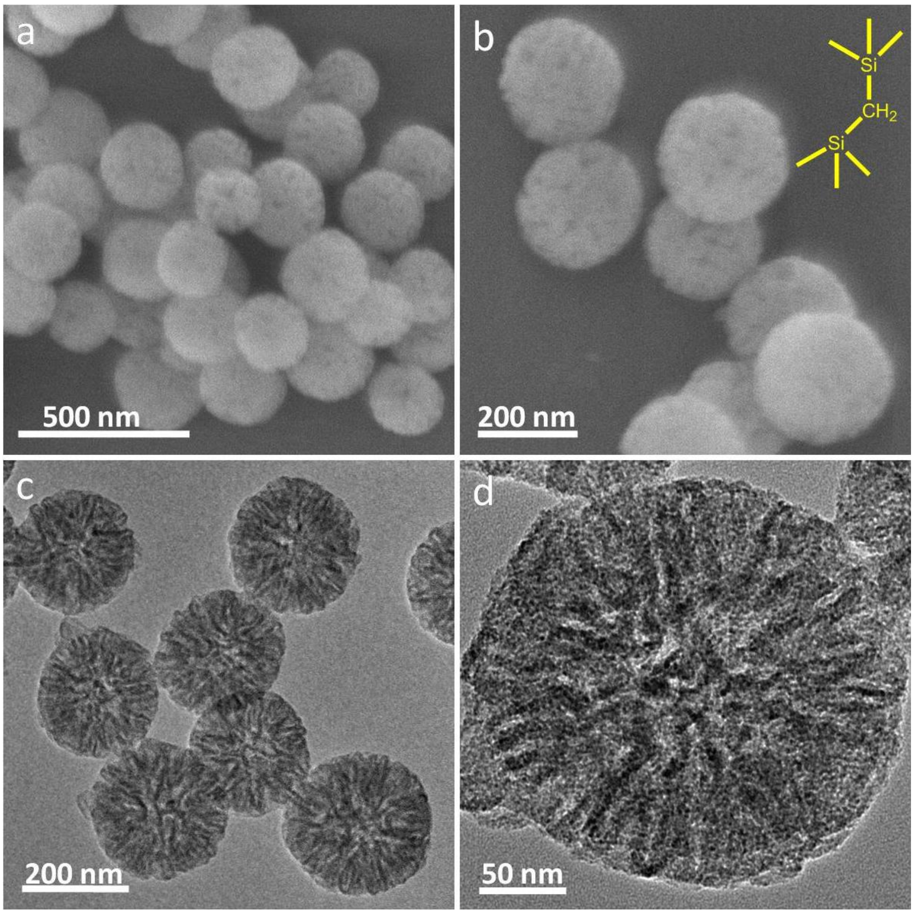

Supplementary Figure 5 | Characterization of MCMOS prepared using organosilane BTME as the precursor. (a, b) SEM and (c, d) TEM images of the MCMOS materials prepared using organosilane BTME as the precursor.

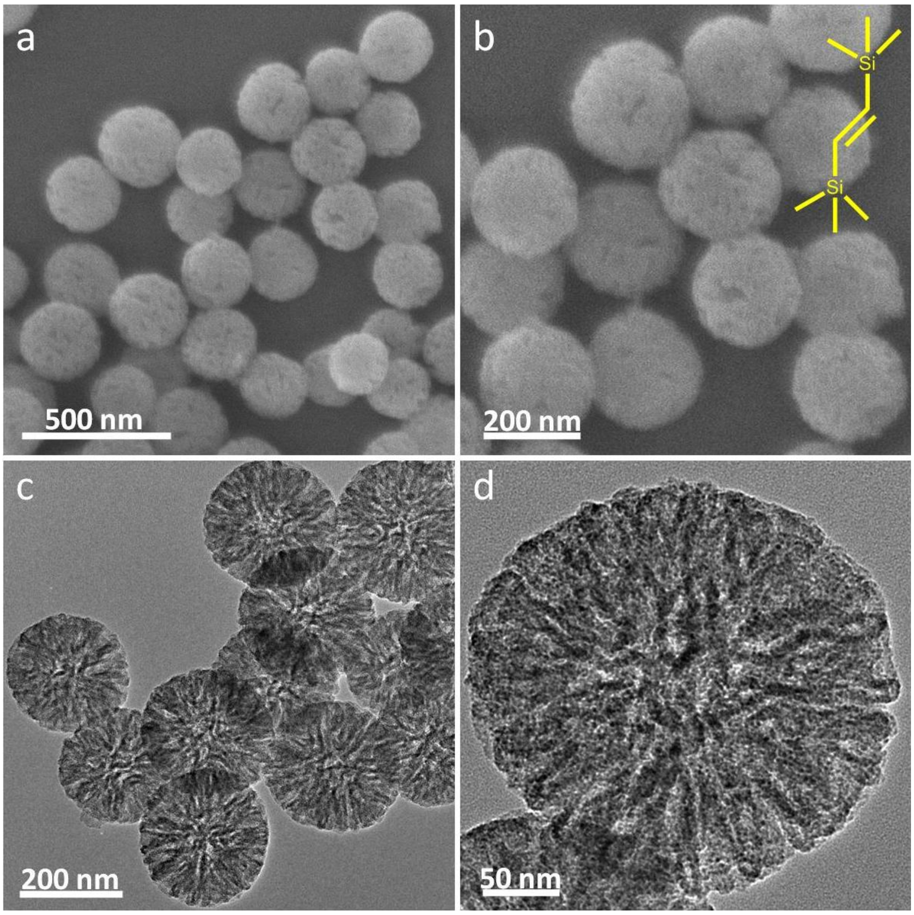

Supplementary Figure 6 | Characterization of MCMOS prepared using organosilane BTEEE as the precursor. (a, b) SEM and (c, d) TEM images of the MCMOS materials prepared using organosilane BTEEE as the precursor.

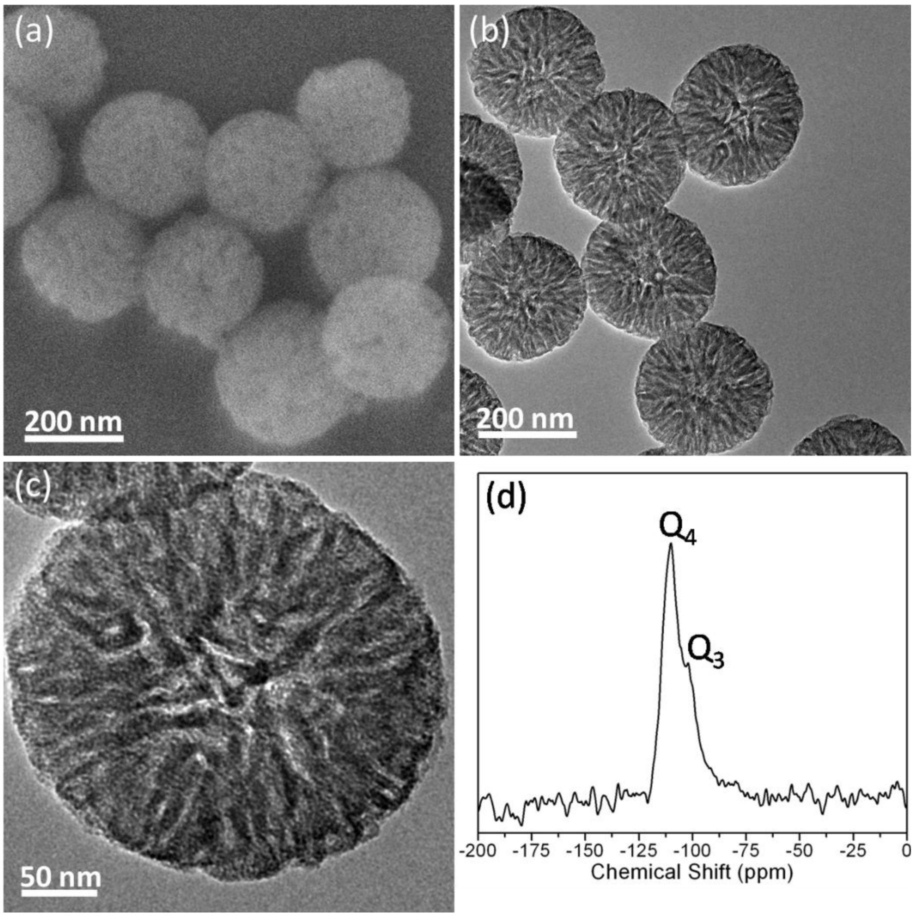

Supplementary Figure 7 | Characterization of the multicompartmentalized mesoporous silica prepared by calcining the MCMOS materials in air at 550 ^{o}C for 6 h. (a) SEM image, (b, c) TEM images and (d) solid state ^{29}Si NMR spectrum of the multicompartmentalized mesoporous silica prepared by calcining the MCMOS materials in air at 550 ^{o}C for 6 h.

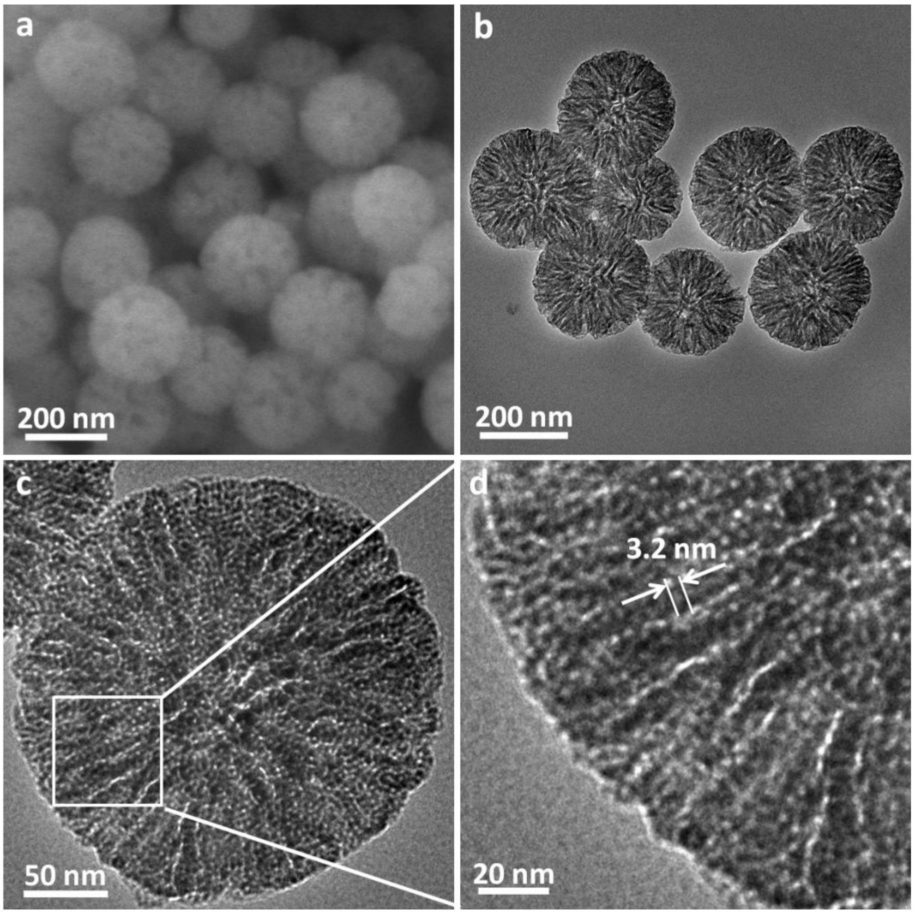

Supplementary Figure 8 | Characterization of MCMOS prepared using OTAC as the surfactant. (a) SEM and (b, c, d) TEM images of the MCMOS materials prepared using octadearyltrimethylammonium chloride (OTAC) as the surfactant under the same conditions for MCMOS prepared using CTAB as the surfactant.

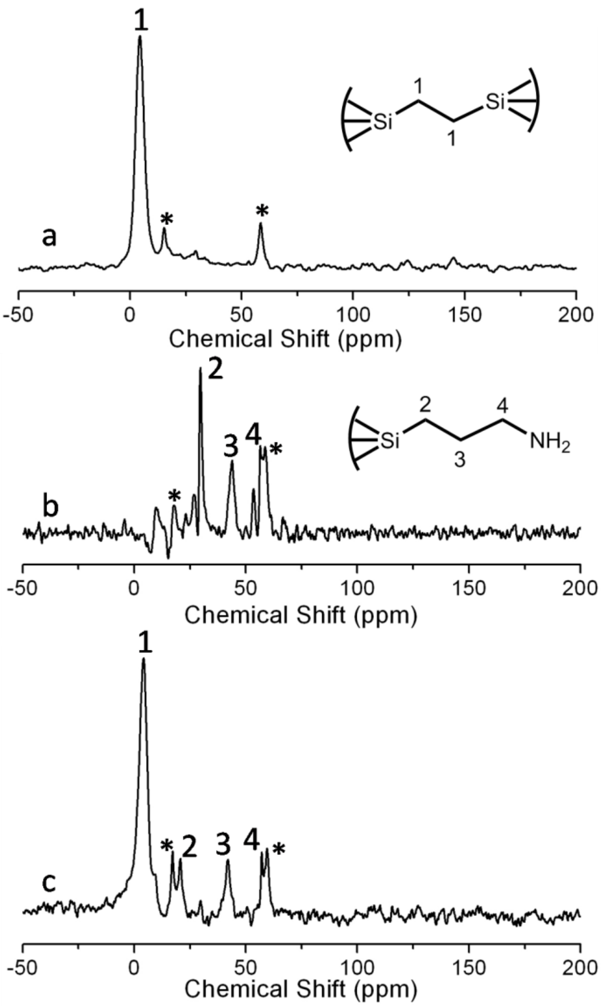

Supplementary Figure 9 | Characterization of MCMOS prepared using aminopropyl group modified DSNs as the hard template. Solid state ^{13}C NMR spectra of (a) MCMOS prepared using DSNs as the hard template, (b) aminopropyl group modified DSNs and (c) MCMOS prepared using aminopropyl group modified DSNs as the hard template.

Noting: The peaks labeled by * could be assigned to the carbon species of residual surfactants inside mesopores[4]. By comparing the solid state ^{13}C NMR spectra of above three samples, it could be concluded that the aminopropyl group has been functionalized on the surface of MCMOS prepared using aminopropyl group modified DSNs as the hard template. The surface aminopropyl group of MCMOS played an important role in stably anchoring Ru NPs and subsequent Pd NPs.

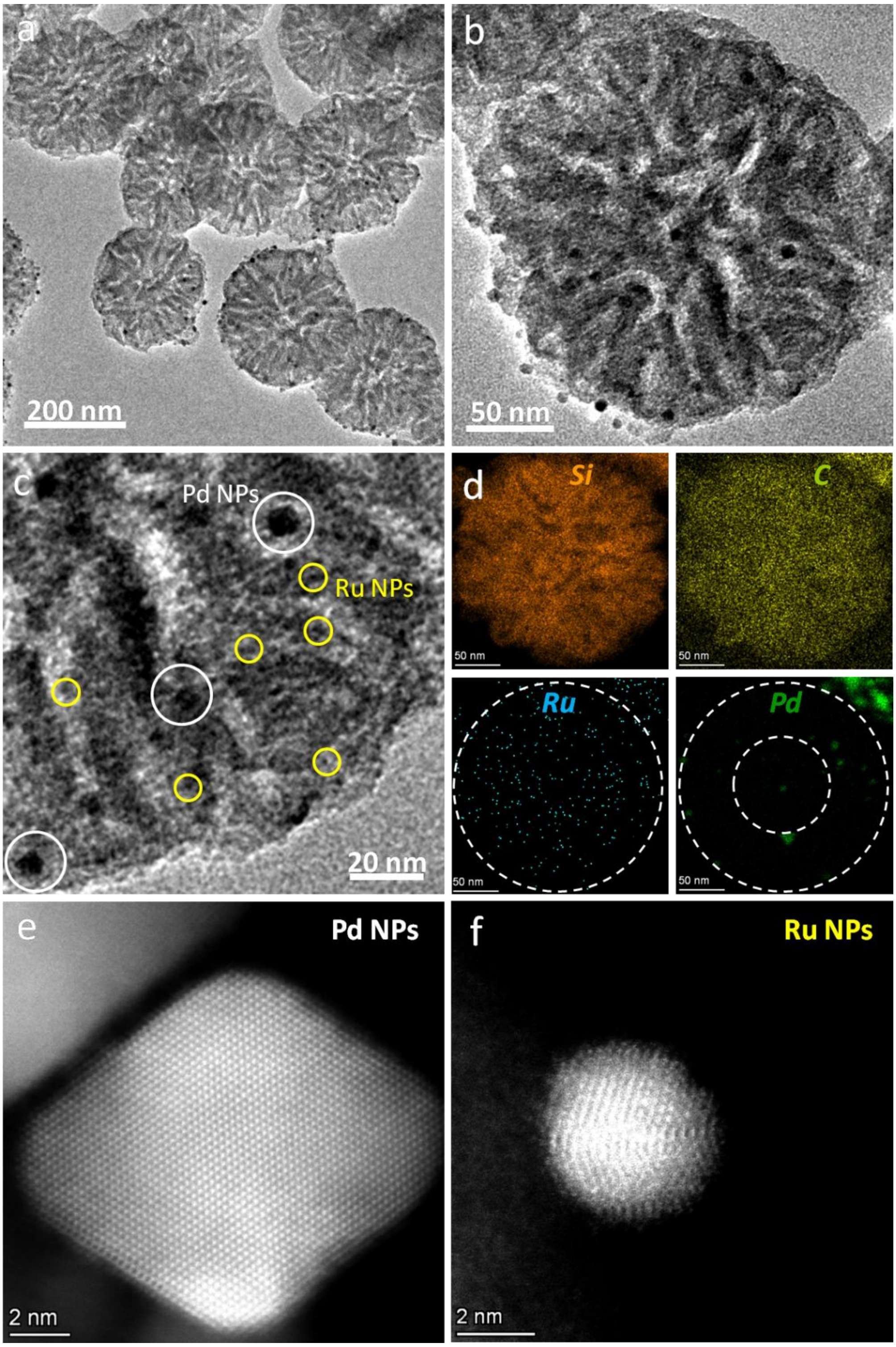

Supplementary Figure 10 | Characterization of the Ru/Pd/MCMOS cascade catalyst. (a, b, c) TEM images and (d) EDX element mapping of the Ru/Pd/MCMOS cascade catalyst. (e, f) Atomic-resolution HAADF-STEM images of Pd NPs (e) and Ru NPs (f) in the Ru/Pd/MCMOS cascade catalyst.

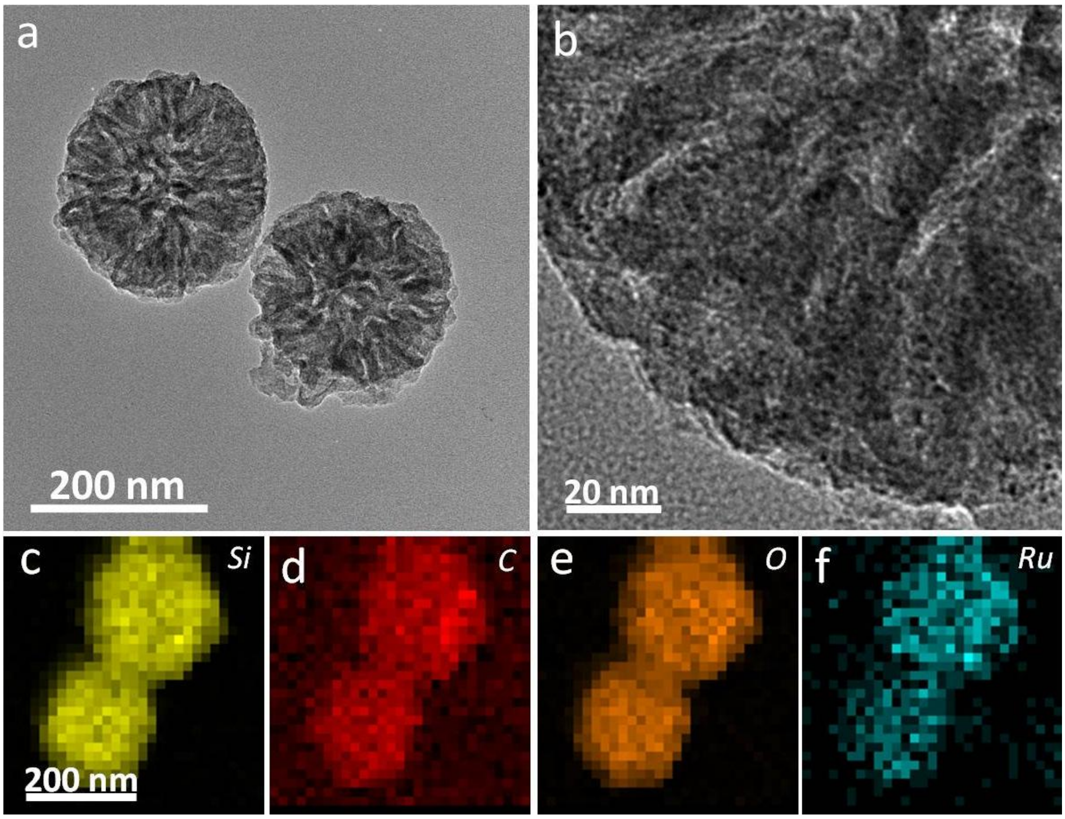

Supplementary Figure 11 | Characterization of the Ru/MCMOS catalyst. (a, b) TEM images and (c, d, e, f) EDX elemental mapping of the monometallic Ru/MCMOS catalyst prepared under the same conditions for the Ru/Pd/MCMOS cascade catalyst except without loading Pd NPs.

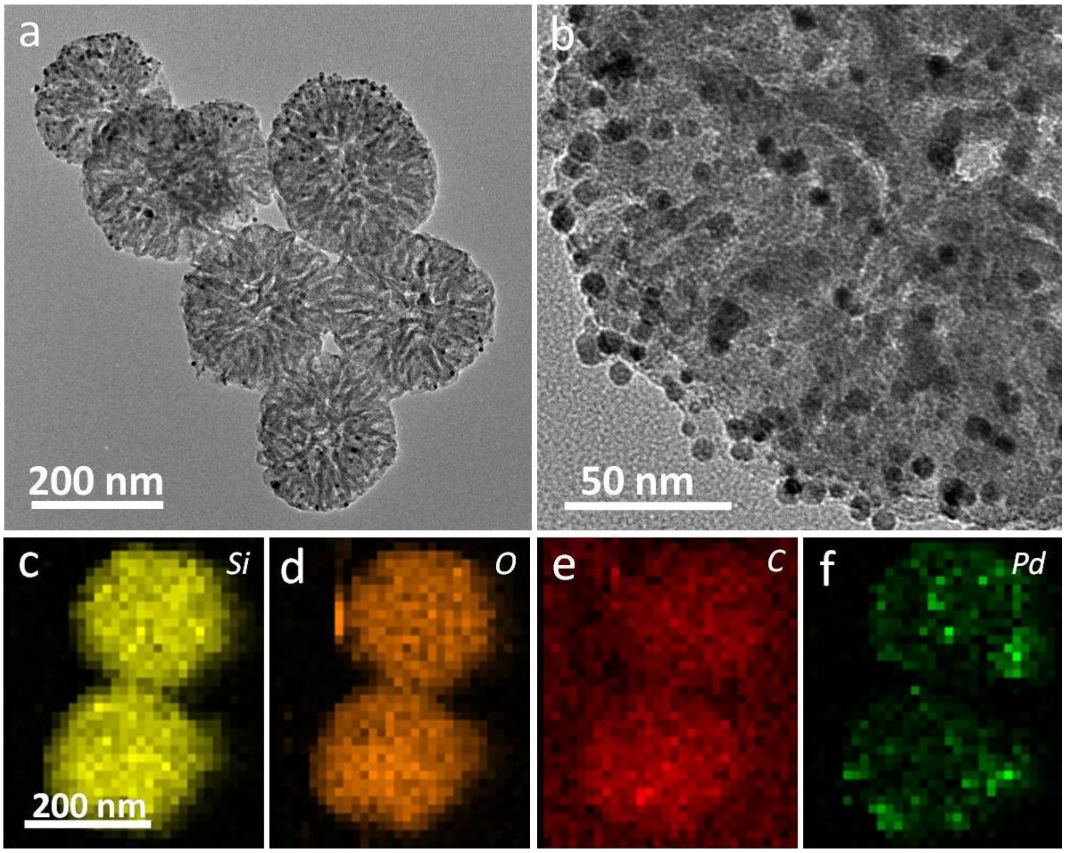

Supplementary Figure 12 | Characterization of the Pd/MCMOS catalyst. (a, b) TEM images and (c, d, e, f) EDX elemental mapping of the monometallic Pd/MCMOS catalyst prepared under the same conditions for the Ru/Pd/MCMOS cascade catalyst except replacing Ru/MCMOS with MCMOS.

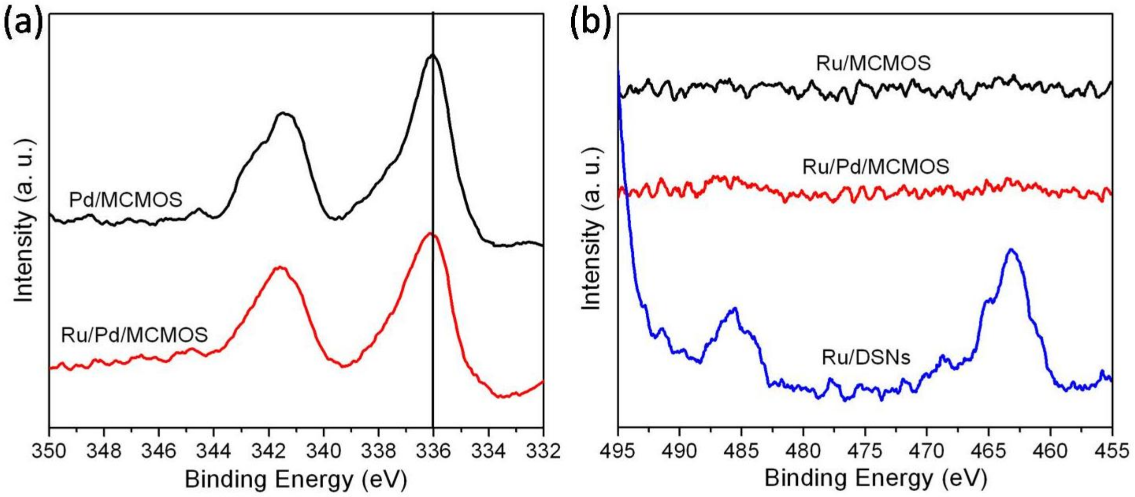

Supplementary Figure 13 | Characterization of the Ru/Pd/MCMOS cascade catalyst. (a) Pd 3*d* XPS spectra of Pd/MCMOS and Ru/Pd/MCMOS. (b) Ru 3*p* XPS spectra of Ru/MCMOS, Ru/Pd/MCMOS and Ru/DSNs.

Noting: According to the XPS analysis, the atomic ratio of Pd/Si of the Ru/Pd/MCMOS catalyst was measured to be nearly 1/37. The ICP-MS analysis revealed that the Pd loading amount of the Ru/Pd/MCMOS catalyst was approximately 0.75 wt%. On the basis of this value, the atomic ratio of Pd/Si of the Ru/Pd/MCMOS catalyst was estimated to be about 1/200. Obviously, the atomic ratio of Pd/Si from the XPS result (1/37) was higher than that from the ICP-MS result (1/200), indicating that the atomic ratio of Pd/Si on the surfaces of the Ru/Pd/MCMOS catalyst was higher than that in bulk phase. This result implied that most of the Pd NPs were loaded on the surface grooves of MCMOS. Different from the results for Pd, we did not detect any Ru signals using XPS analysis, proving that the Ru NPs have been encapsulated inside the interior nanocavity of MCMOS. XPS results provided evidence about the spatial separation of Ru NPs and Pd NPs in the Ru/Pd/MCMOS cascade catalyst.

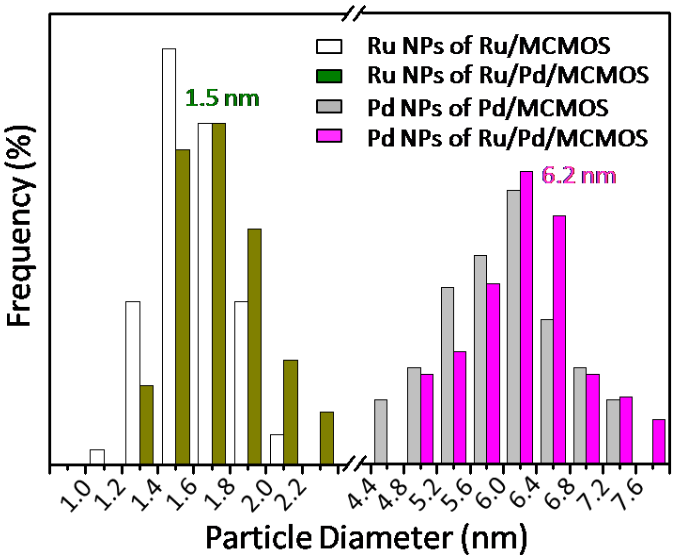

Supplementary Figure 14 | Characterization of the Ru/Pd/MCMOS cascade catalyst. Particle size distributions for the Ru NPs and the Pd NPs of Ru/MCMOS, Pd/MCMOS and Ru/Pd/MCMOS.

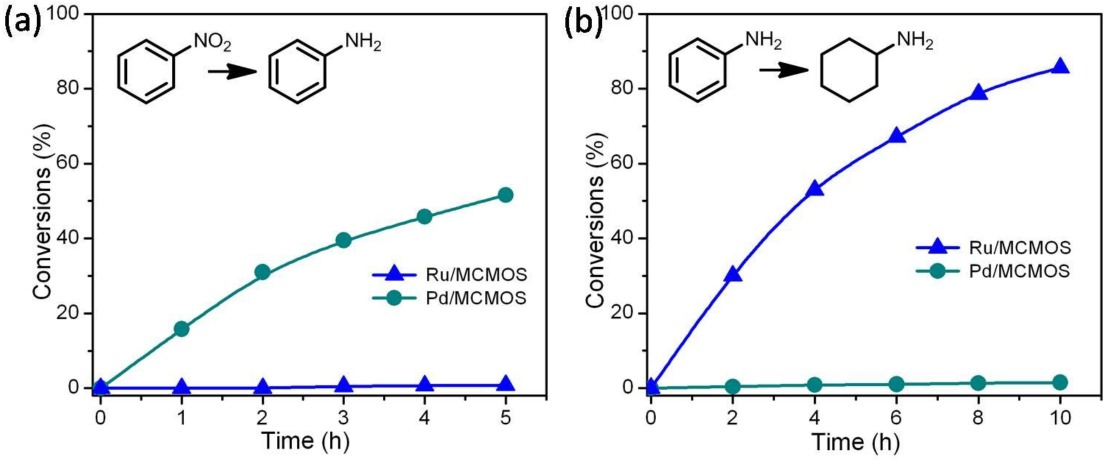

Supplementary Figure 15 | Catalytic performances evaluation. (a) Kinetic plots of the hydrogenation of nitrobenzene to aniline (the first step) over two monometallic catalysts: Ru/MCMOS (triangle) and Pd/MCMOS (circle). (b) Kinetic plots of the hydrogenation of aniline to cyclohexylamine (the second step) over two monometallic catalysts: Ru/MCMOS (triangle) and Pd/MCMOS (circle). Reaction conditions: nitrobenzene or aniline (0.25 mmol), solid catalyst (Ru 0.26 mol% or Pd 0.34 mol%), ethanol (2.0 mL), H_2 (2.0 MPa), 80 ^oC.

Noting: In order to precisely test the hydrogenation kinetics of both of steps, the used doage of catalysts was decreased to 33% to slow down the step reactions in comparison to the sequential hydrogenation reaction.

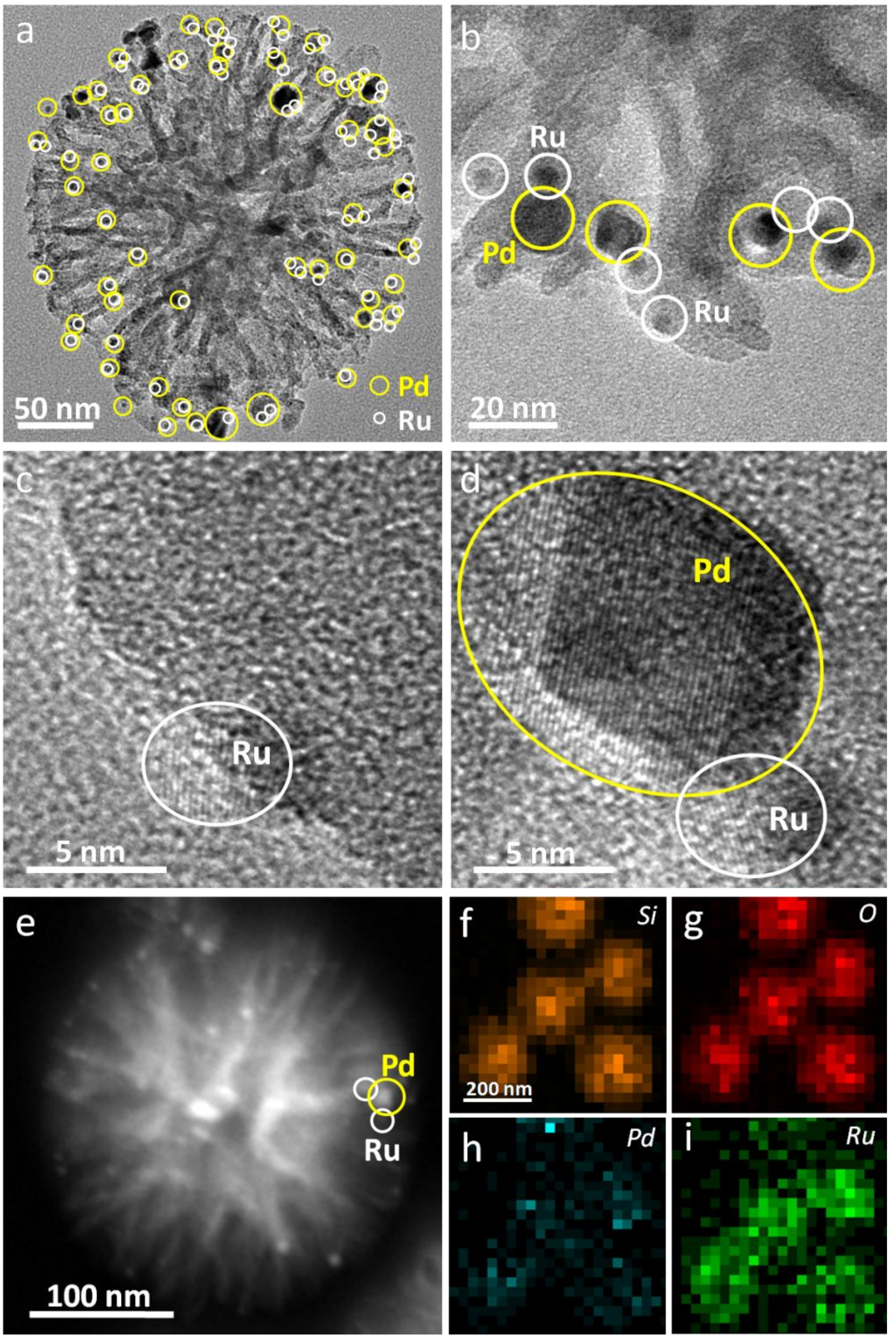

Supplementary Figure 16 | Characterization of the Ru-Pd/DSNs catalyst. (a, b) TEM images, (c, d) RHTEM images, (e) HAADF-STEM image and (f, g, h, i) EDX elemental mapping of the Ru-Pd/DSNs catalyst with the Pd and Ru NPs jointly loaded inside the channels of DSNs.

Noting: By randomly counting more than 100 nanoparticles, we found that ≥ 90% of Pd nanoparticles have more than one Ru nanopaticles on their surfaces. The HRTEM image in Supplementary Fig. 16c indicates that there are only a little of free Ru nanopaticles being located in the channels of DSNs. The HRTEM image in Figure S16d clearly indicates that the Ru nanopaticles is exactly located on the surface of Pd nanoparticles.

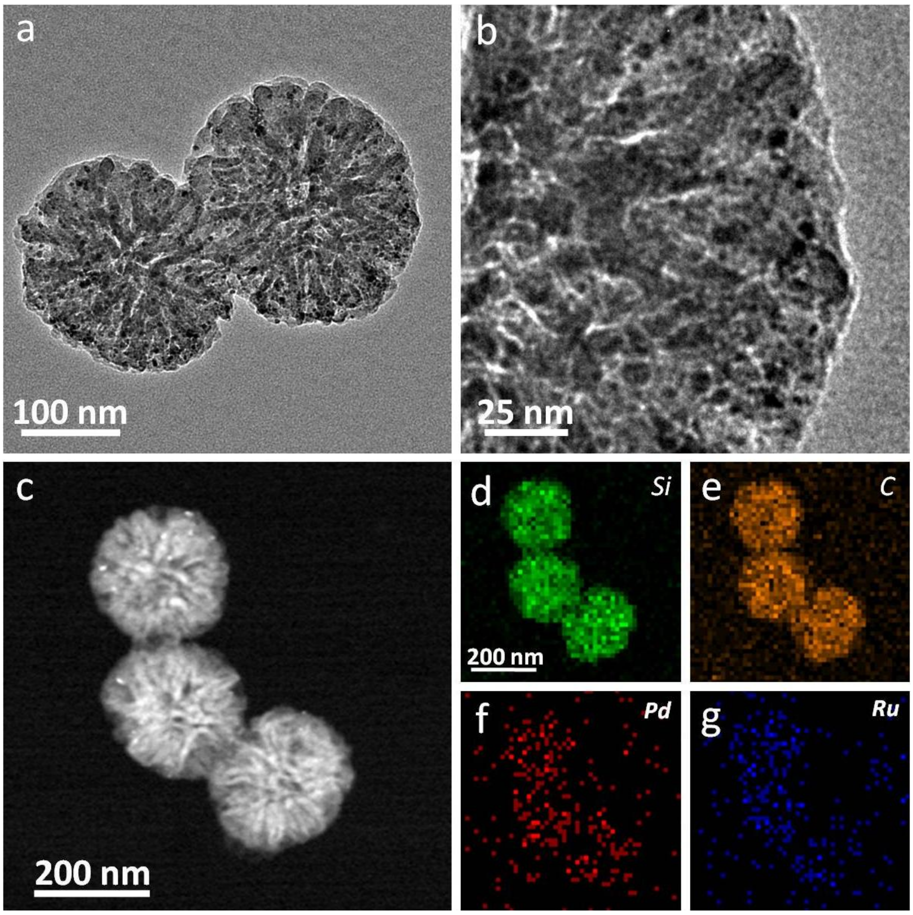

Supplementary Figure 17 | Characterization of the PdRu/MCMOS alloy catalyst. (a, b) TEM images, (c) HAADF-STEM image and (d, e, f, g) EDX elemental mapping of the PdRu/MCMOS alloy catalyst with the PdRu solid solution alloy NPs loading inside the MCMOS.

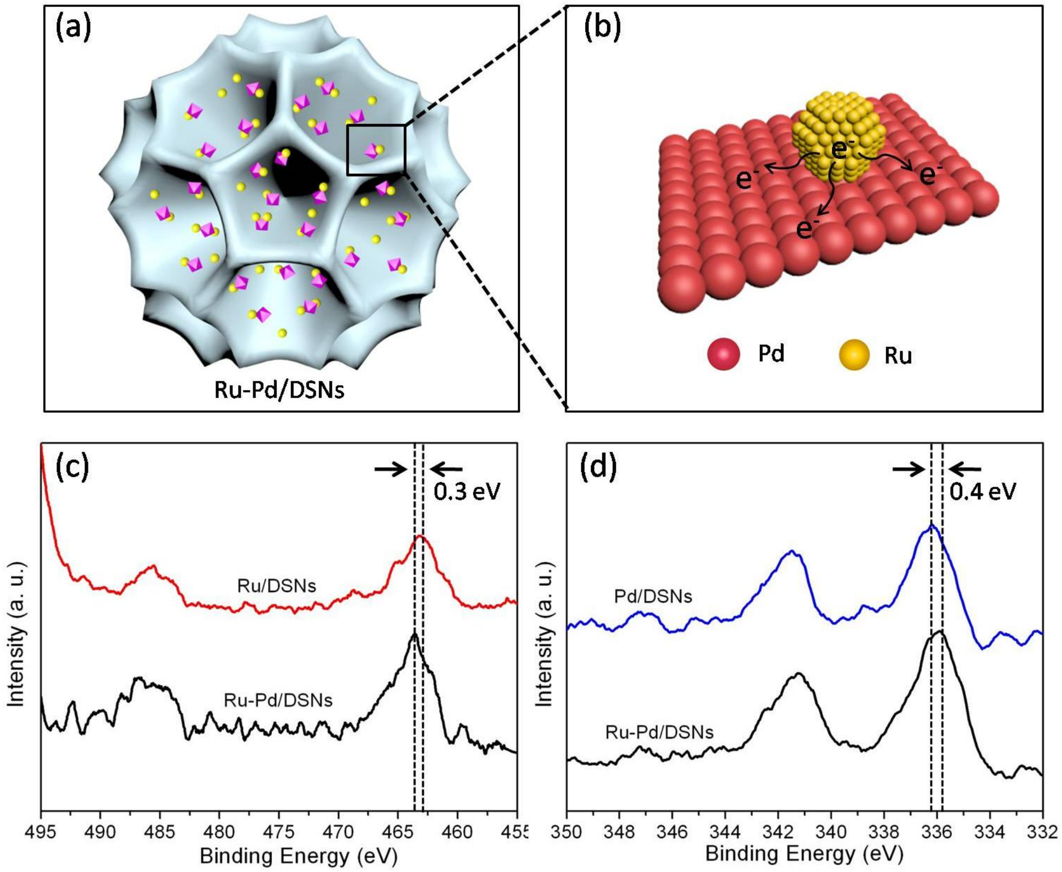

Supplementary Figure 18 | Characterization of the Ru-Pd/DSNs catalyst. (a) Illustration of the Ru-Pd/DSNs catalyst. (b) Illustration of the possible charge transfer process when the small Ru NPs and the large Pd NPs are jointly loaded in the dendritic channels of DSNs (small Ru NPs are around the large Pd NPs, see TEM images in Supplementary Fig. 16). (c) Ru 3*p* XPS spectra of Ru/DSNs and Ru-Pd/DSNs. (d) Pd 3*d* XPS spectra of Pd/DSNs and Ru-Pd/DSNs.

Noting: As displayed in Supplementary Fig. 18c, the Ru $3p_{3/2}$ binding energy of Ru-Pd/DSNs was obviously blue-shifted from 463.2 eV to 463.5 eV in comparison to that of Ru/DSNs. Meanwhile, compared with the Pd $3d_{5/2}$ peak position of Pd/DSNs, the Pd $3d_{5/2}$ binding energy of Ru-Pd/DSNs was obviously red-shifted from 336.2 eV to 335.8 eV. Because of the identical conditions for loading metal NPs, the Ru particle size of Ru-Pd/DSNs was as same as that of Ru/DSNs and the Pd particle size of Ru-Pd/DSNs remained in good agreement with that of Pd/DSNs. Since the disturbance of metal particle size could be excluded, such a distinct shift of binding energy in both Ru 3*p* and Pd 3*d* XPS spectra provided clear evidences for the charge transfer from Ru to Pd in the Ru-Pd/DSNs catalyst.

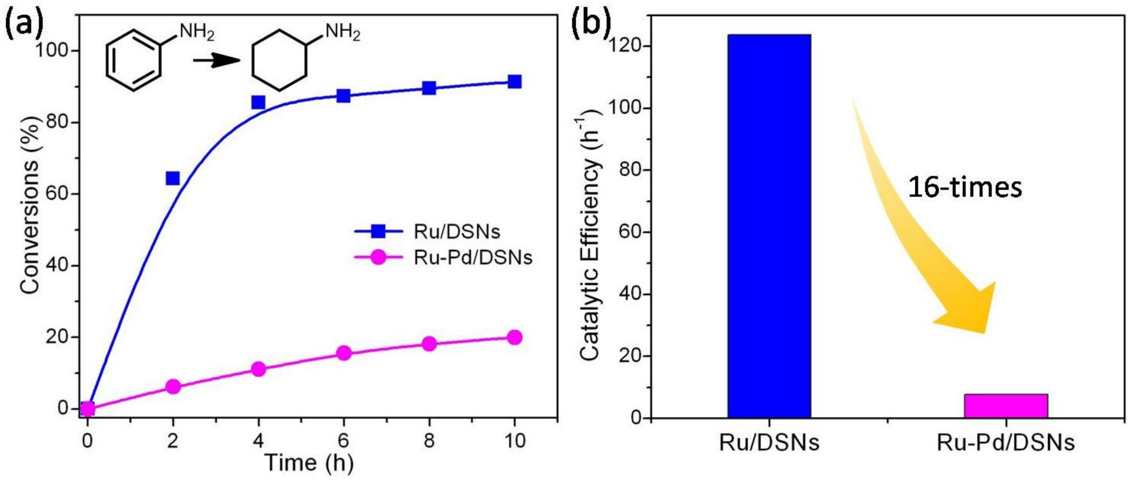

Supplementary Figure 19 | Catalytic performances evaluation. (a) Kinetic plots of the hydrogenation of aniline to cyclohexylamine (the second step) over different catalysts: Ru/DSNs (square) and Ru-Pd/DSNs (sphere). (b) Catalytic efficiencies for the Ru/DSNs catalyst and the Ru-Pd/DSNs catalyst. Catalytic efficiency is defined as the moles of converted reactants per mole of catalyst per hour (mol mol^{-1} h^{-1}), calculated using low conversions ($\leq$ 20%). Reaction conditions: aniline (0.25 mmol), solid catalyst (Ru 0.26 mol%, Pd 0.34 mol%), ethanol (2.0 mL), H_2 (2.0 MPa), 80 ^oC.

Noting: In comparison to the monometallic Ru/DSNs catalyst, the catalytic activity of the Ru-Pd/DSNs catalyst decreases up to 16-fold in the hydrogenation of aniline to cyclohexylamine (the second step). Such a distinct deactivation indicates that there is a negative effect for the dual metal NPs in the Ru-Pd/DSNs catalyst without spatial separation. The XPS results in Supplementary Fig. 18 reveal that charge transfer from Ru to Pd decreases the surface electron density of Ru NPs, which weakens the activation of aromatic ring on the surfaces of Ru NPs[5-6]. Such a serious deactivation demonstrated that the Ru NPs and the Pd NPs were incompatible in the second step of sequential hydrogenation reactions due to the electronic effects.

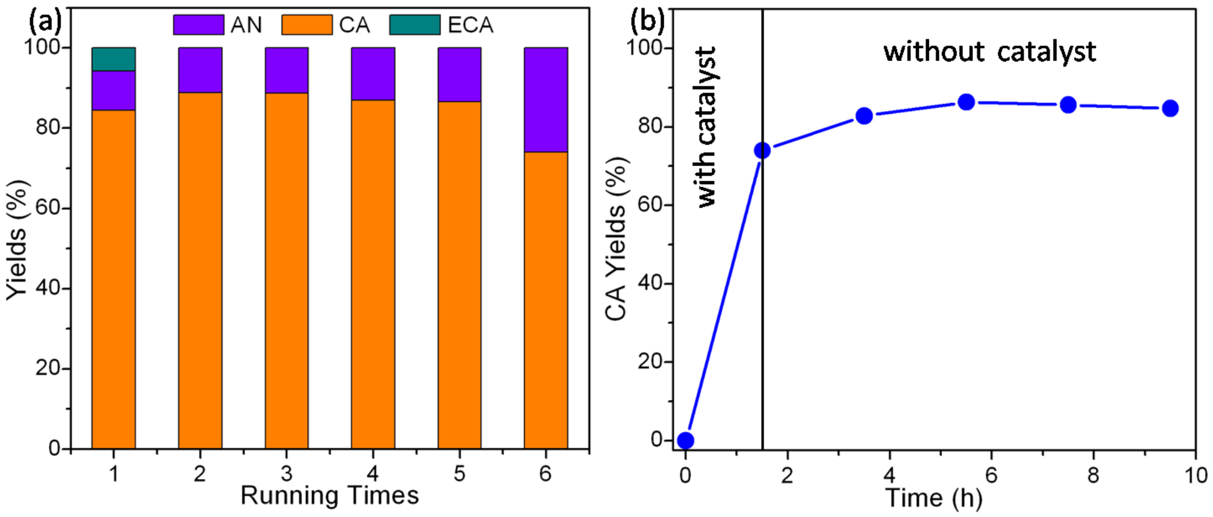

Supplementary Figure 20 | Catalytic recyclability of Ru/Pd/MCMOS. (a) Recyclability of the Ru/Pd/MCMOS catalyst in the sequential hydrogenation of nitrobenzene to cyclohexylamine. (b) The reaction profile for the filtrate test (Reaction condition: after the sixth reaction cycle proceeded for 1.5 h, the catalyst was removed from the reaction system by centrifugation and the residual filtrate was allowed for further reaction under the same conditions. All other conditions were consistent with the process of reaction shown in Fig. 3).

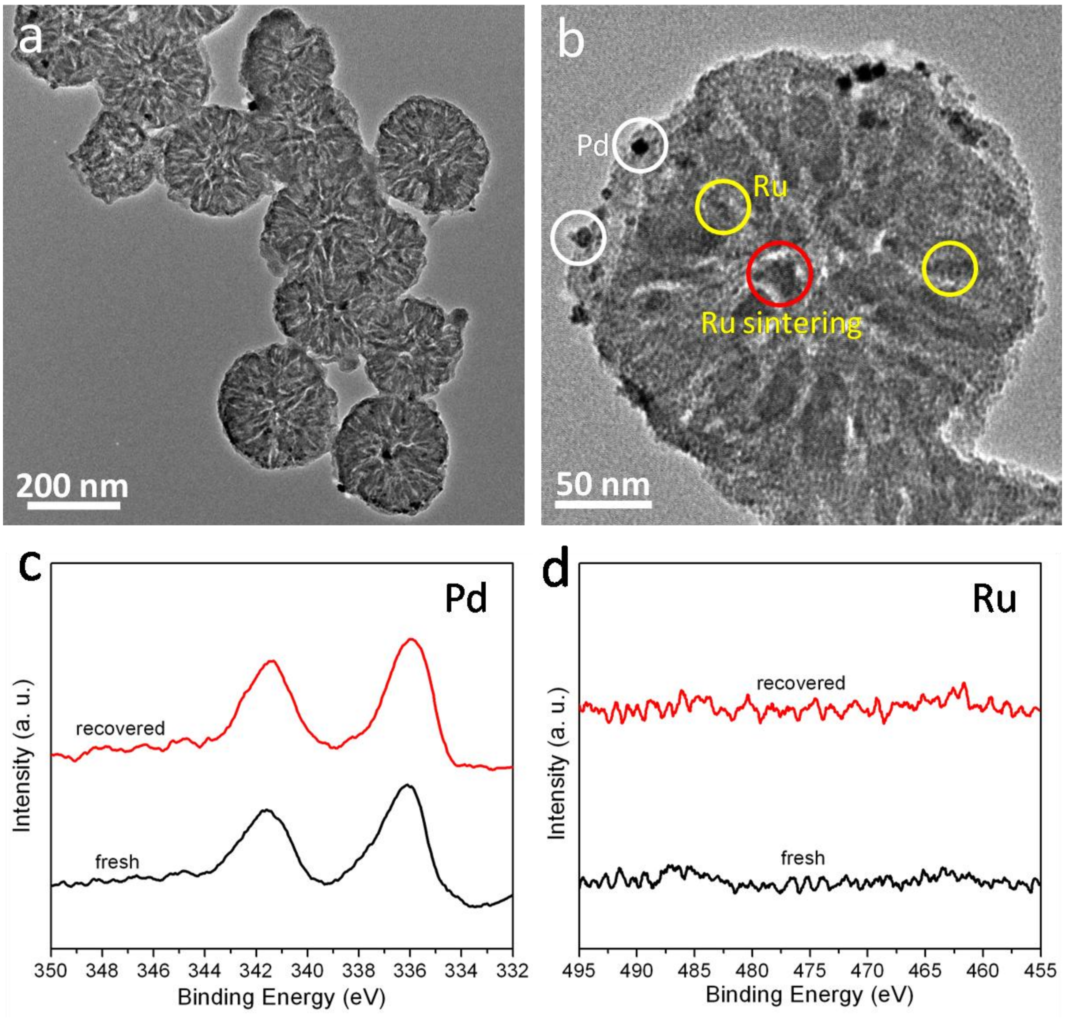

Supplementary Figure 21 | Characterization of the recovered Ru/Pd/MCMOS catalyst. (a-b) TEM images, (c) Pd 3*d* XPS spectrum and (d) Ru 3*p* XPS spectrum of the recovered Ru/Pd/MCMOS catalyst.

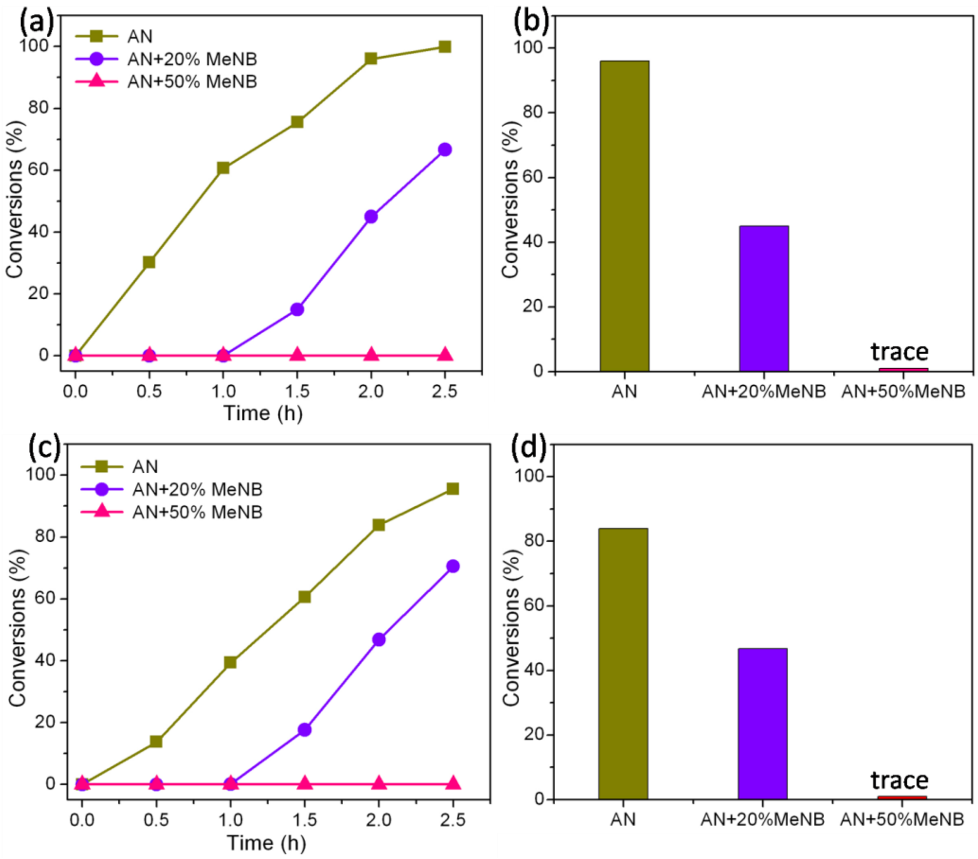

Supplementary Figure 22 | Substrate inhibition effect. (a) Kinetic plots of aniline hydrogenation over the Ru/MCMOS catalyst in presence of different amounts of p-nitrotoluene. (b) Conversions of aniline for the Ru/MCMOS catalyst within 2.0 h in presence of different amounts of p-nitrotoluene (MeNB). (c) Kinetic plots of aniline hydrogenation over the Ru/MCMOS catalyst pre-reduced at 200 ^{o}C in presence of different amounts of p-nitrotoluene. (d) Conversions of aniline for the Ru/MCMOS catalyst pre-reduced at 200 ^{o}C within 2.0 h in presence of different amounts of p-nitrotoluene (MeNB). Reaction conditions: aniline (0.25 mmol), solid catalyst (Ru 0.78 mol%), ethanol (2.0 mL), H_2 (2.0 MPa), 80 ^{o}C.

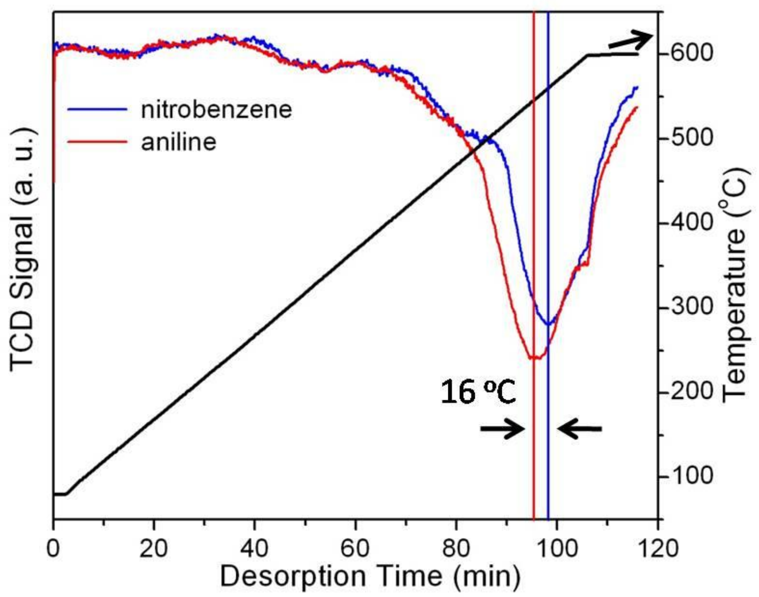

Supplementary Figure 23 | Substrate inhibition effect. Nitrobenzene-TPD profile and aniline-TPD profile of the Ru/MCMOS catalyst.

Noting: The desorption temperature of nitrobenzene molecules on the Ru/MCMOS catalyst was 560 ^{o}C, which was higher than that of aniline molecules (544 ^{o}C). Although parts of chemisorbed nitrobenzene and aniline have been possibly decomposed at such high desorption temperatures, the higher desorption/decomposition temperature for adsorbed nitrobenzene still suggested stronger adsorption than aniline. This result indicated that nitrobenzene molecules preferentially adsorbed on the Ru NPs of the Ru/MCMOS catalyst in comparison to aniline molecules. The preferential adsorption of nitrobenzene blocked the surface active sites for the adsorption of aniline on the Ru NPs, resulting in the substrate inhibition effect observed on the Ru NPs. Thus only when the nitrobenzene has been fully converted into aniline, the aniline started to be transformed to cyclohexylamine over the Ru NPs. This was further evidenced by the reaction kinetics of sequential hydrogenation over the Ru/Pd/MCMOS cascade catalyst (Fig. 3e).

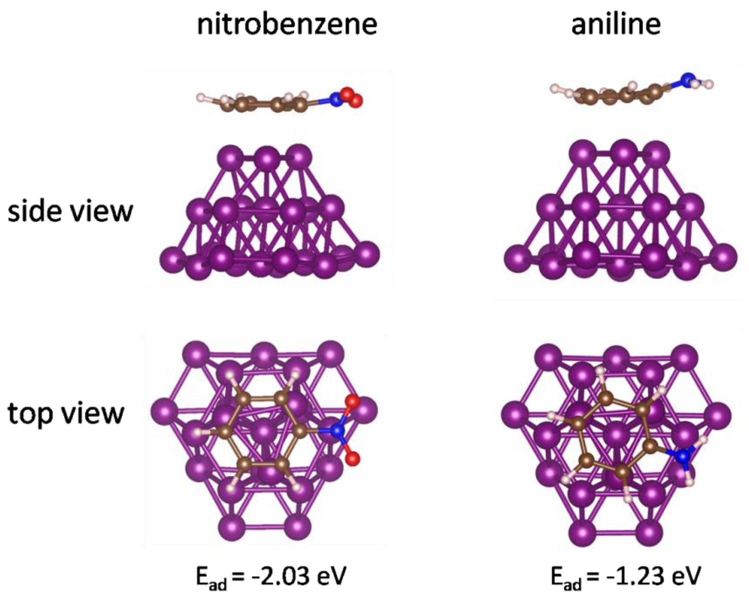

Supplementary Figure 24 | Substrate inhibition effect. The adsorption structures and adsorption energies of nitrobenzene and aniline on the Ru_{22} cluster.

Noting: Density functional theory calculations were performed to further investigate the adsorption of nitrobenzene and aniline on the Ru/MCMOS catalyst. Ru_{22} cluster that was close to the Ru NPs of Ru/MCMOS in particle size was used as a model to carry out the calculation. As shown in the Supplementary Figure 24, both the nitrobenzene molecule and the aniline molecule preferentially bind with the Ru_{22} cluster via aromatic ring due to the strong d-π_{aryl} interaction. The adsorption energy of nitrobenzene molecule on the Ru_{22} cluster is -2.03 eV, much higher that than of aniline molecule (-1.23 eV). This result indicates that the binding interaction of nitrobenzene molecule on the Ru_{22} cluster is stronger than the aniline molecules. This implies that nitrobenzene molecules would preferentially adsorb on the Ru NPs of the Ru/MCMOS catalyst in comparison to aniline molecules. The preferential adsorption of nitrobenzene blocks the surface active sites for the adsorption of aniline on the Ru NPs, resulting in the substrate inhibition effect observed on the Ru NPs.

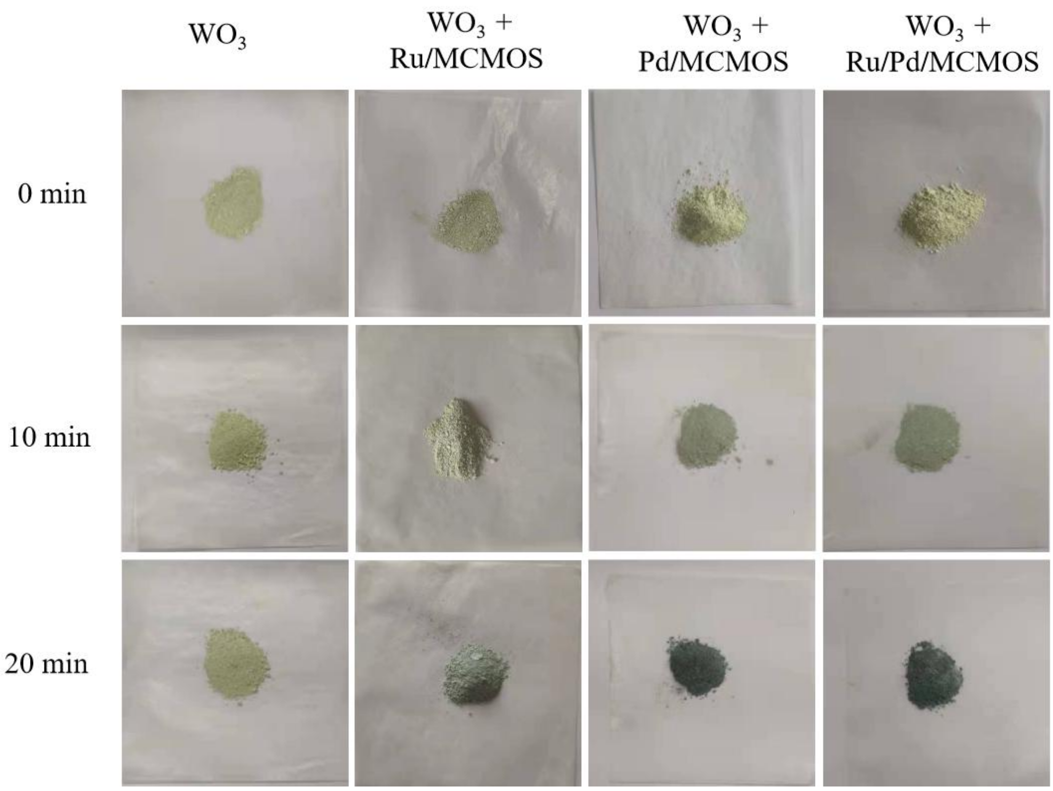

Supplementary Figure 25 | Hydrogen spillover between different catalysts and WO_3. Photographs of samples made with 0.5 g of WO_3 mixed with 0.01 g of different catalysts after treatment with H_2 at 80 ℃ for different times.

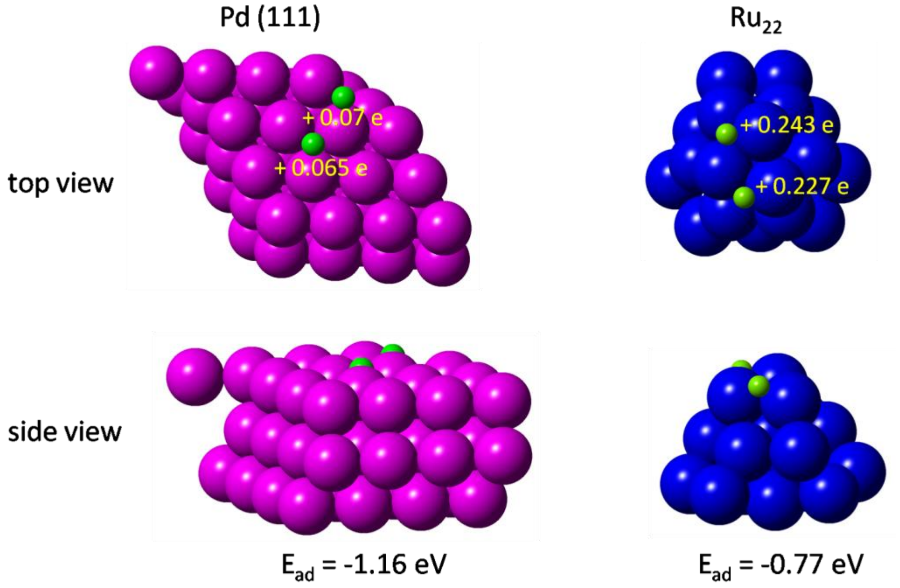

Supplementary Figure 26 | Hydrogen activation on different catalysts. The adsorption structures and adsorption energies of H_2 molecule on the Pd (111) surface and the Ru_{22} cluster. In both cases, both of H atoms get similar electrons (highlighted in the structure of top view) after the dissociation of H_2 molecule.

Noting: As for H_2 activation, homolytic dissociation into H atoms and heterolytic dissociation into H^+/H^- pairs represent the two most common pathways. Because noble metals (Pd, Pt, Rh and Ru) possessed superior capacity for H_2 activation, which resulted from their partially occupied d-orbitals, homolytic dissociation of H_2 to active H atoms commonly occurs on the surfaces of noble metal nanoparticles. To confirm the activation pathways of H_2, density functional theory (DFT) calculations were conducted to examine the adsorption of H_2 on the simplified model Pd (111) surface and Ru_{22} cluster. As shown in Supplementary Fig. 25, dissociative adsorption of H_2 molecule was observed on both of models. The adsorption energy of H_2 molecule on the Pd (111) surface and Ru_{22} cluster was -1.16 eV and -0.77 eV, respectively. Importantly, both of H atoms on the Pd (111) surface get similar electrons (0.07 Vs 0.065) after the dissociation of H_2 molecule, and a similar result was also observed on the Ru_{22} cluster. These results indicate that homolytic dissociation of H_2 have occurred on the surfaces of Pd NPs and Ru NPs to generate active hydrogen atoms for the hydrogention reactions.

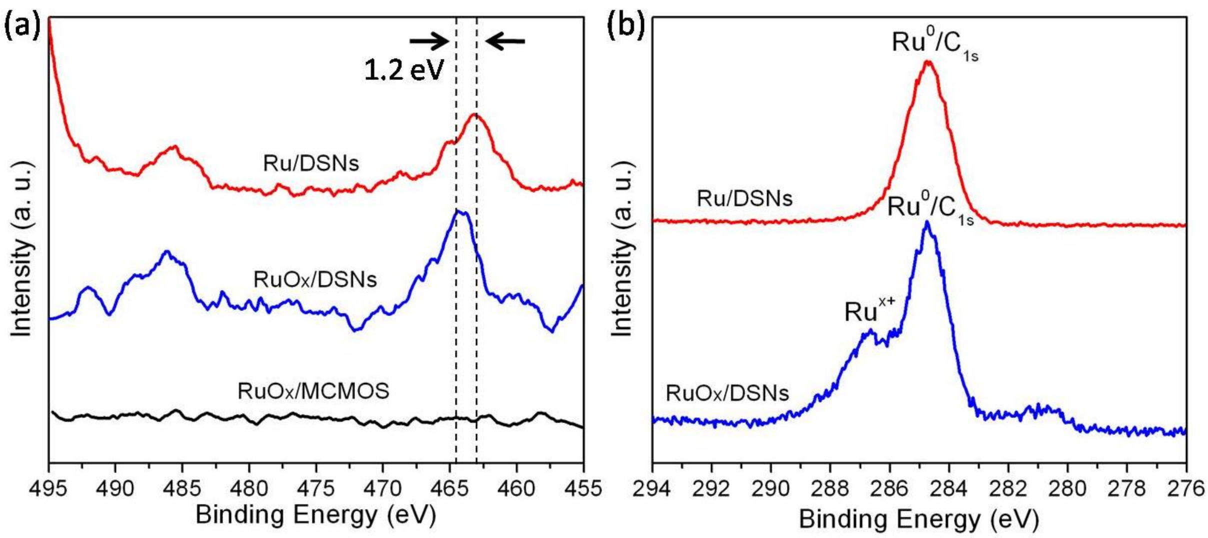

Supplementary Figure 27 | Characterization of RuO_x/MCMOS. (a) Ru 3*p* XPS spectra of Ru/DSNs, RuO_x/DSNs and RuO_x/MCMOS. (b) Ru 3*d* XPS spectra of Ru/DSNs and RuO_x/DSNs. RuO_x/DSNs and RuO_x/MCMOS were respectively prepared by oxidizing Ru/DSNs and Ru/MCMOS using hypochlorous acid.

Noting: In comparison to Ru/DSNs, the Ru 3*p* binding energy of RuO_x/DSNs is positively shifted, suggesting that the Ru NPs on DSNs have been partly oxidized after being treated by hypochlorous acid.[7] No obvious Ru 3*p* peaks appear in the XPS spectrum of RuO_x/MCMOS, indicating that the RuO_x species are still confined within the nanocavities of MCMOS. Moreover, Ru oxidation state species can be clearly observed in the 3*d* XPS spectrum of RuO_x/DSNs.

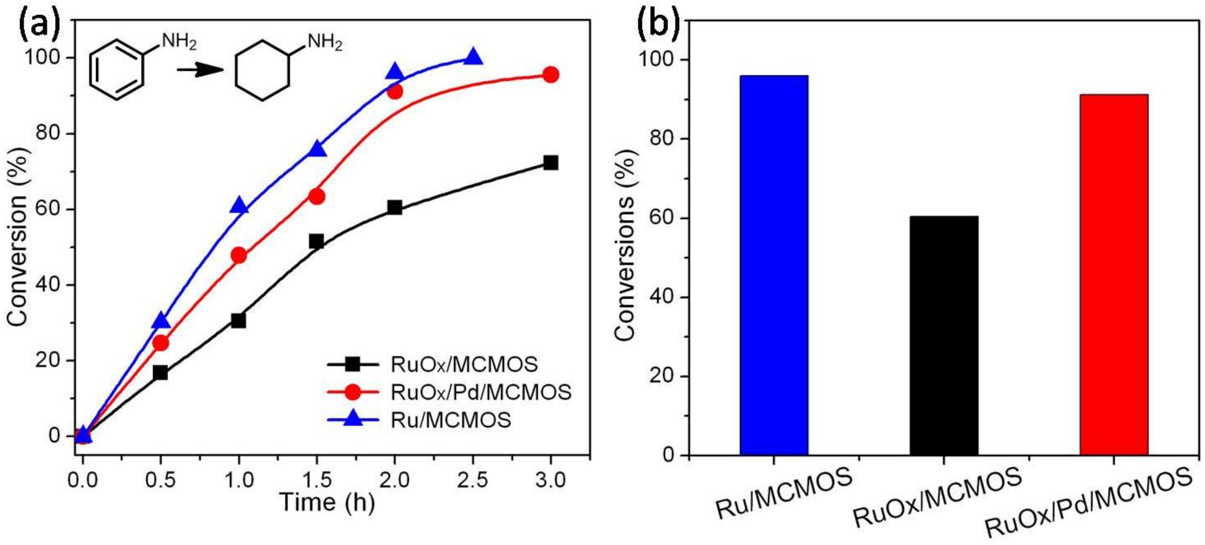

Supplementary Figure 28 | Catalytic performances evaluation. (a) Kinetic plots of aniline hydrogenation over RuO_x/MCMOS, Ru/MCMOS and RuO_x/Pd/MCMOS. (b) Conversions of aniline for RuO_x/MCMOS, Ru/MCMOS and RuO_x/Pd/MCMOS within 2 h. Reaction conditions: aniline (0.25 mmol), solid catalyst (Ru 0.78 mol%), ethanol (2.0 mL), H_2 (2.0 MPa), 80 ^{o}C.

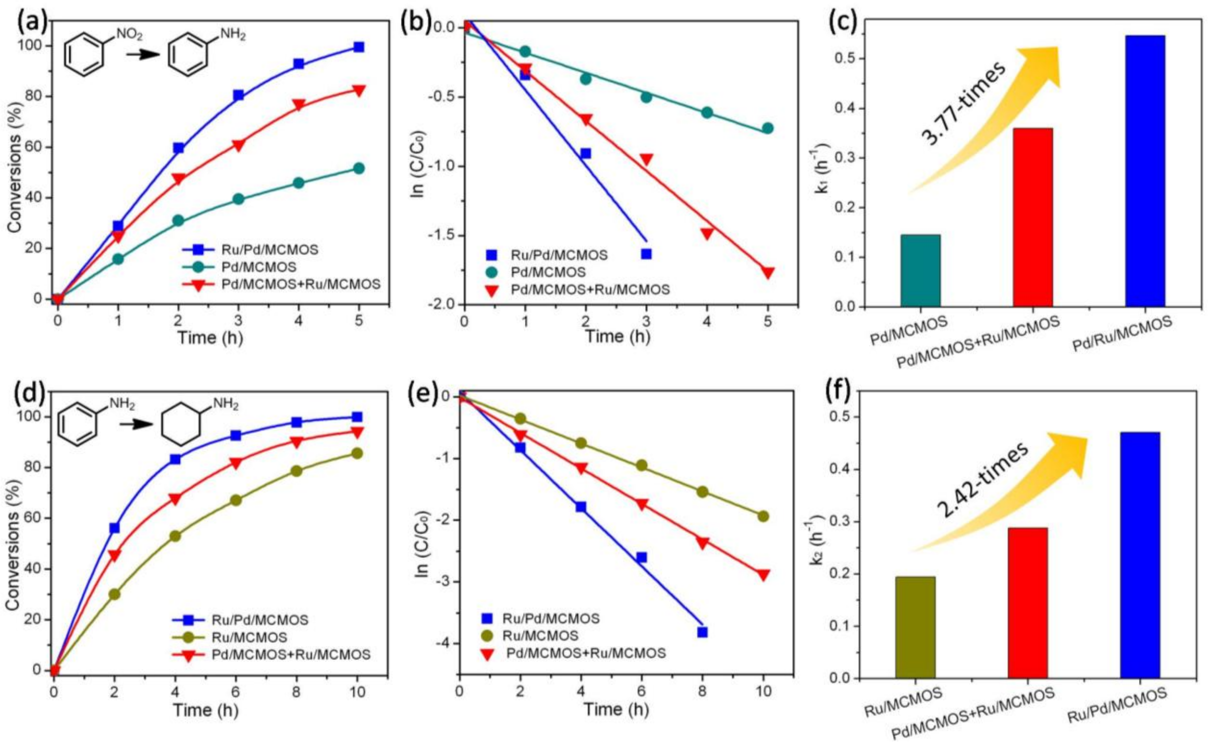

Supplementary Figure 29 | Catalytic performances evaluation. (a) Kinetic plots of the hydrogenation of nitrobenzene to aniline (the first step) over different catalysts: Pd/MCMOS (circle), Ru/Pd/MCMOS (square) and the physically mixed catalyst (triangle). (b) Plot of time vs $\ln(C/C_{0,\,NB})$ for the first step over different catalysts. (c) The corresponding rate constant value (k_1) of the first step for different catalysts. (d) Kinetic plots of the hydrogenation of aniline to cyclohexylamine (the second step) over different catalysts: Ru/MCMOS (triangle) Ru/Pd/MCMOS (square) and the physically mixed catalyst (triangle). (e) Plot of time vs $\ln(C/C_{0,\,AN})$ for the second step over different catalysts. (f) The corresponding rate constant value (k_2) of the second step for different catalysts. Reaction conditions: nitrobenzene or aniline (0.25 mmol), solid catalyst (Ru 0.26 mol% or Pd 0.34 mol%), ethanol (2.0 mL), H_2 (2.0 MPa), 80 ^{o}C.

Noting: By plotting $\ln(C/C_0)$ as a function of reaction time, we found that there was a nearly linear relationship between $\ln(C/C_0)$ and the reaction time for all the catalysts in both of hydrogenation reactions. This observation indicated that all these reactions followed pseudo first-order kinetics. Therefore, the rate constants of two catalytic steps (k_1 and k_2) can be obtained from the slope in accordance to the rate equation $\ln(C/C_0) = kt$. Additionally, in order to precisely test the hydrogenation kinetic of both of steps, the used doage of catalysts was decreased to 33% to slow down the step reactions in comparison to the sequential hydrogenation reaction.

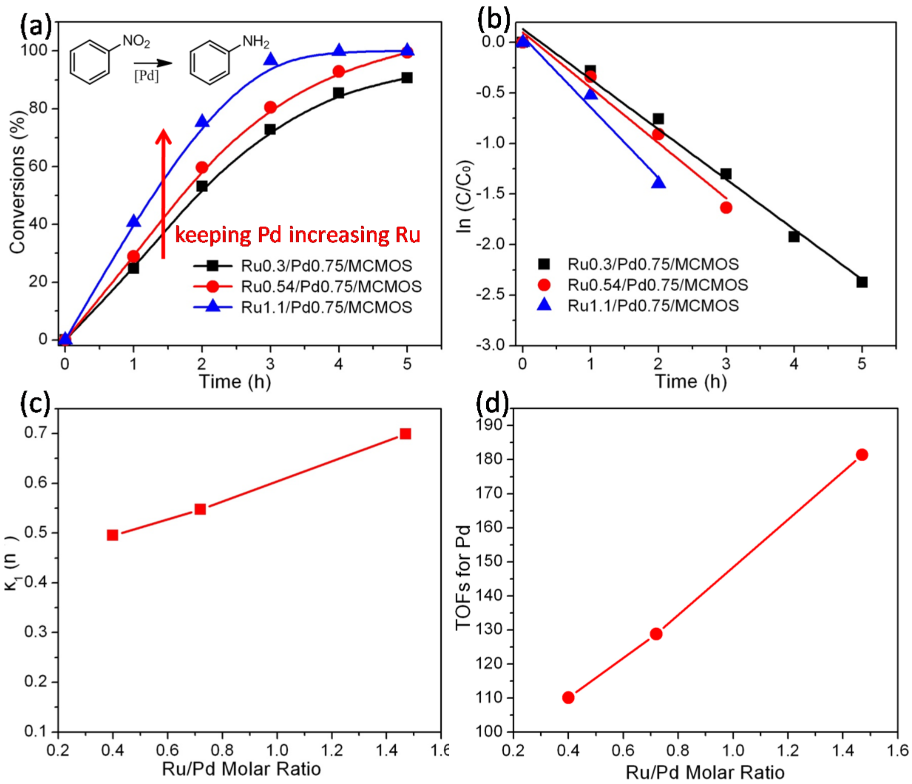

Supplementary Figure 30 | Catalytic performances evaluation. (a) Kinetic plots of the hydrogenation of nitrobenzene to aniline (the first step) over the Ru/Pd/MCMOS cascade catalysts with constant Pd loading yet different Ru loadings. (b) Plot of time vs ln($C/C_{0,\ NB}$) for the first step over the Ru/Pd/MCMOS catalysts with different Ru/Pd ratios. (c) The corresponding rate constant value (k_1) of the first step for the Ru/Pd/MCMOS catalysts with different Ru/Pd ratios. (d) Turnover frequencies (TOFs) of the metal Pd of the Ru/Pd/MCMOS catalysts with different Ru/Pd ratios. Reaction conditions: nitrobenzene (0.25 mmol), solid catalyst (Pd 0.34 mol%), ethanol (2.0 mL), H_2 (2.0 MPa), 80 ^{o}C.

Noting: Since the Ru/Pd/MCMOS catalysts with different Ru/Pd ratios were prepared by loading the same Pd NPs (beforehand prepared) in the Ru/MCMOS with different Ru loadings, we believed that the Pd dispersion of the Ru/Pd/MCMOS catalysts could be considered approximately equal to that of the monometallic Pd/MCMOS catalyst. Thus the Pd dispersion of the Ru/Pd/MCMOS catalysts was obtained from the H_2-TPD data of Pd/MCMOS. Then the TOFs of Pd for the Ru/Pd/MCMOS catalysts were calculated according to their reaction kinetics (the first step). As shown in Supplementary Fig. 30d, the TOFs of Pd for the Ru/Pd/MCMOS catalysts increased from 110.1 h^{-1} to 181.4 h^{-1} when increasing the Ru/Pd ratio from 0.3/0.75 to 1.1/0.75. This result indicated that the catalytic activity of the metal Pd was enhanced in the hydrogenation of nitrobenzene to aniline with the increasing Ru loading. This is because the presence of Ru NPs did not alter the Pd surface sites of the Ru/Pd/MCMOS catalysts but they supplied active hydrogen to accelerate the hydrogenation reaction on the Pd NPs.

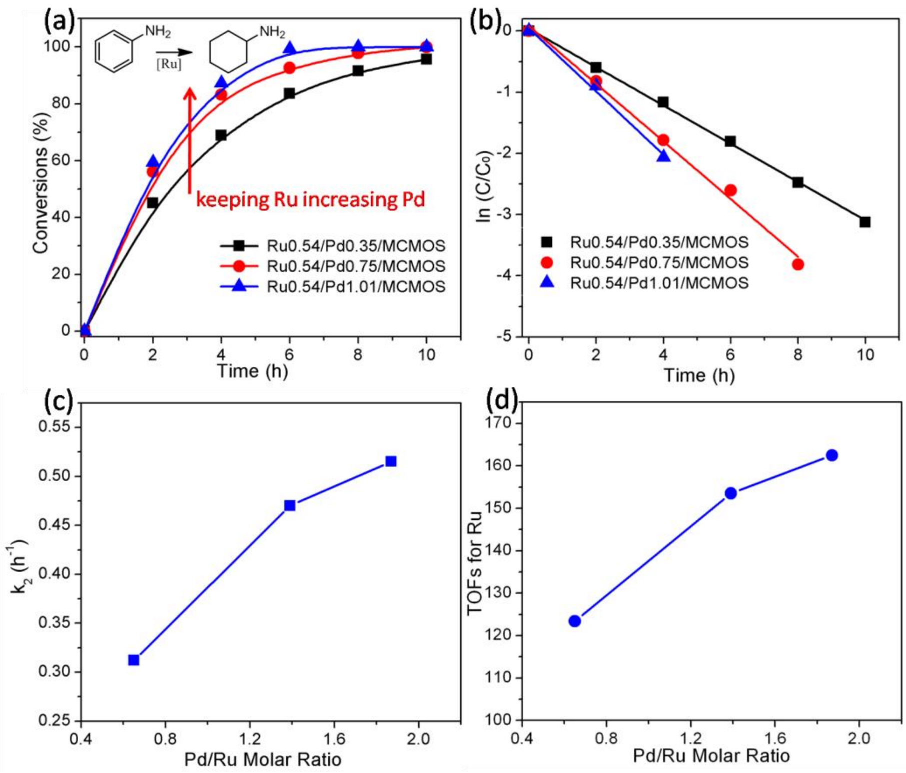

Supplementary Figure 31 | Catalytic performances evaluation. (a) Kinetic plots of the hydrogenation of aniline to cyclohexylamine (the second step) over the Ru/Pd/MCMOS cascade catalysts with constant Ru loading yet different Pd loadings. (b) Plot of time vs ln($C/C_{0,\ AN}$) for the second step over the Ru/Pd/MCMOS catalysts with different Ru/Pd ratios. (c) The corresponding rate constant value (k_2) of the second step for the Ru/Pd/MCMOS catalysts with different Ru/Pd ratios. (d) Turnover frequencies (TOFs) of the metal Ru of the Ru/Pd/MCMOS catalysts with different Pd/Ru ratios. Reaction conditions: aniline (0.25 mmol), solid catalyst (Ru 0.26 mol%), ethanol (2.0 mL), H_2 (2.0 MPa), 80 ^oC.

Noting: Likewise, the Ru dispersion of the Ru/Pd/MCMOS catalysts was obtained from the H_2-TPD data of Ru/MCMOS. Then the TOFs of Ru for the Ru/Pd/MCMOS catalysts were calculated according to their reaction kinetics of converting aniline to cyclohexylamine (the second step). As shown in Supplementary Fig. 31d, the TOFs of Ru for the Ru/Pd/MCMOS catalysts increased from 123.4 h^{-1} to 162.5 h^{-1} when increasing the Pd/Ru ratio from 0.35/0.54 to 1.01/0.54. The presence of Pd NPs did not alter the Ru surface sites of the Ru/Pd/MCMOS catalysts but they supplied active hydrogen to accelerate the hydrogenation reaction on the Ru NPs, leading to the enhanced catalytic activity of the metal Ru in the hydrogenation of nitrobenzene to aniline.

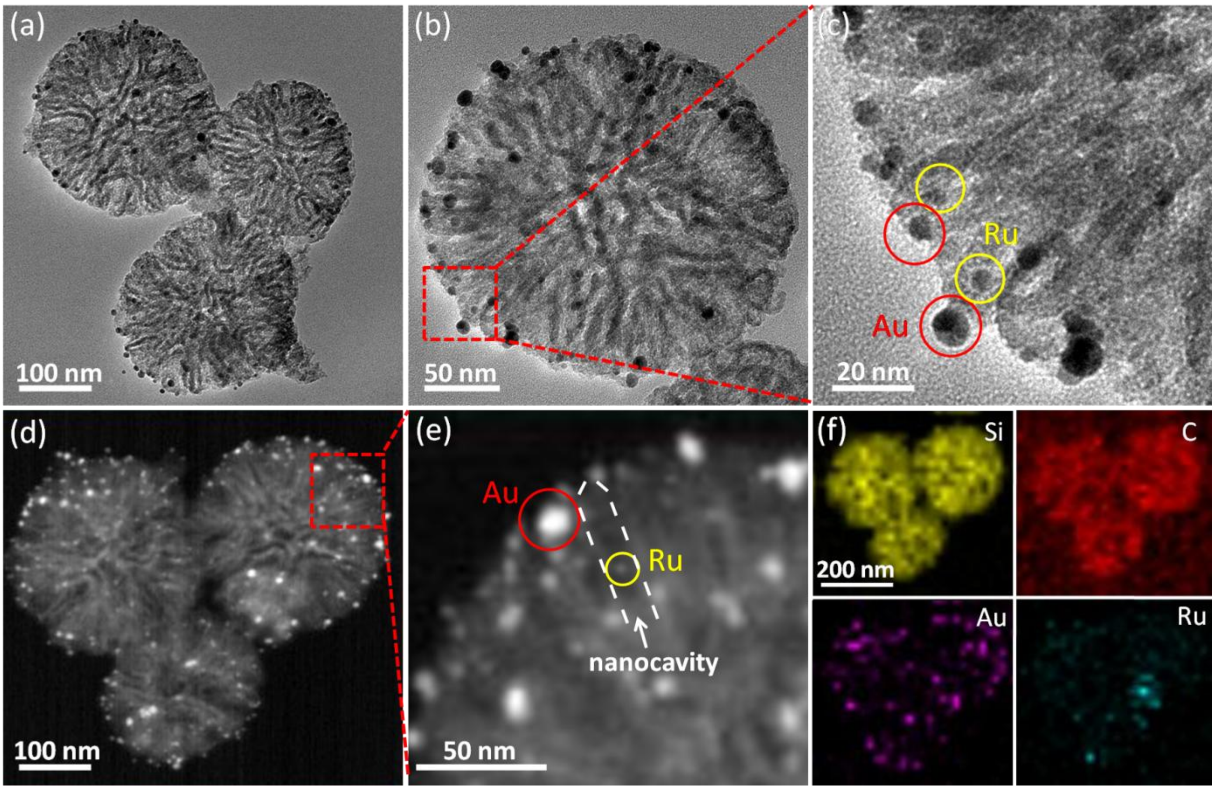

Supplementary Figure 32 | Characterization of the Ru/Au/MCMOS cascade catalyst. (a, b, c) TEM images, (d, e) HAADF-STEM images and (f) EDX element mapping of the Ru/Au/MCMOS cascade catalyst.

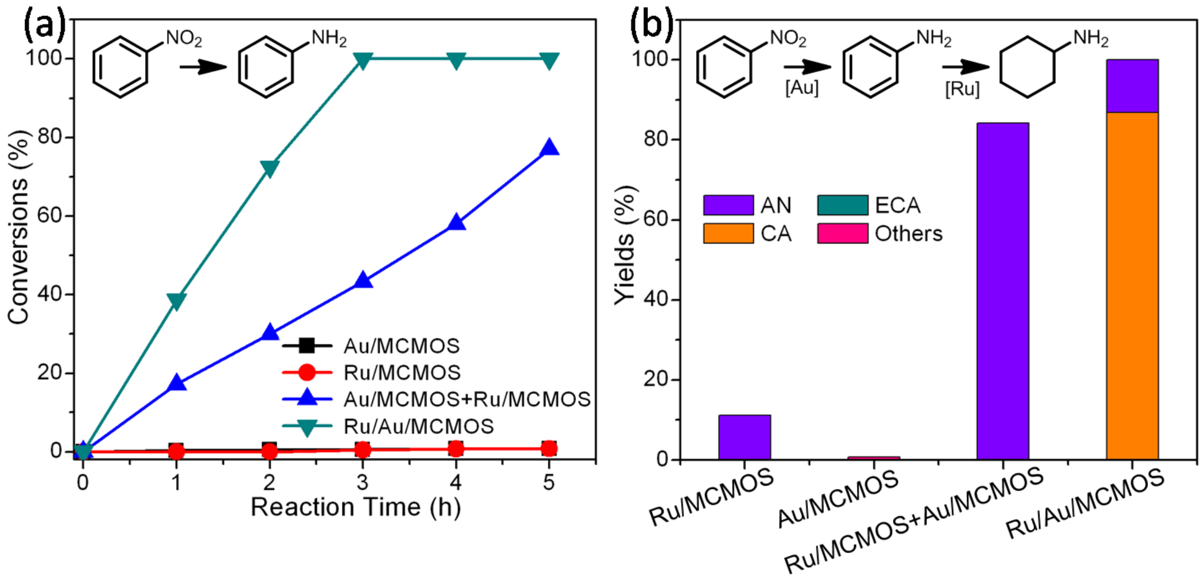

Supplementary Figure 33 | Catalytic performances evaluation. (a) Kinetic plots of the hydrogenation of nitrobenzene to aniline (the first step) over different catalysts: Au/MCMOS (square), Ru/MCMOS (circle), physical mixture of Au/MCMOS and Ru/MCMOS (uptriangle) and Ru/Au/MCMOS (downtriangle). Reaction conditions: nitrobenzene (0.25 mmol), solid catalyst (Ru 0.26 mol% or Au 0.34 mol%), ethanol (2.0 mL), H_2 (2.0 MPa), 80 ^oC. (b) Yields of the sequential hydrogenation over different catalysts: Au/MCMOS, Ru/MCMOS, physical mixture of Au/MCMOS and Ru/MCMOS and Ru/Au/MCMOS. Reaction conditions: nitrobenzene (0.25 mmol), solid catalyst (Ru 0.77 mol% or Au 1.0 mol%), ethanol (2.0 mL), H_2 (2.0 MPa), 80 ^oC.

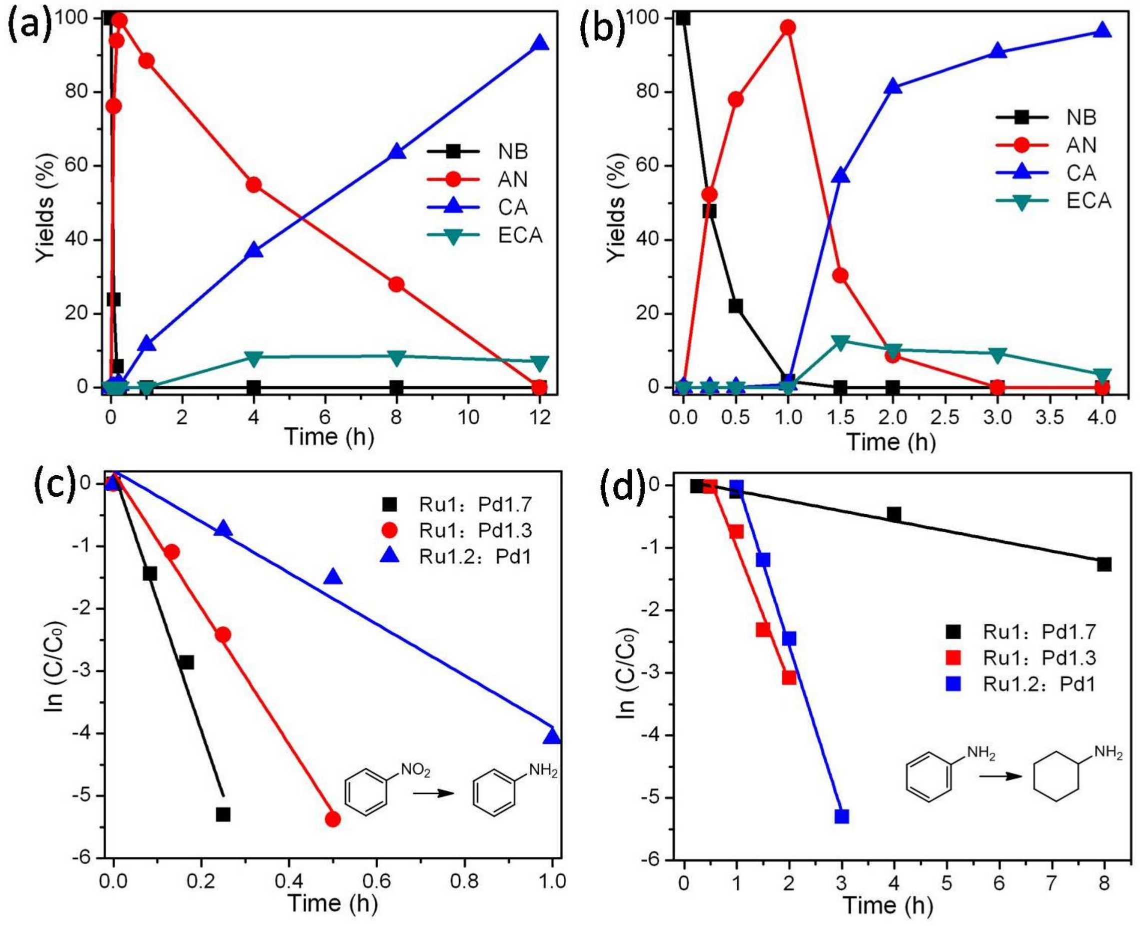

Supplementary Figure 34 | Catalytic performances evaluation. (a, b) Kinetic plots of the sequential hydrogenation of nitrobenzene to cyclohexylamine over the Ru/Pd/MCMOS cascade catalyst with different Ru/Pd ratios: (a) $Ru_1/Pd_{1.7}$/MCMOS and (b) $Ru_{1.2}/Pd_1$/MCMOS. (c) Plot of time vs ln($C/C_{0,\ NB}$) for the first step of the sequential hydrogenation reaction over the Ru/Pd/MCMOS cascade catalysts with different Ru/Pd ratios. (d) Plot of time vs ln($C/C_{0,\ AN}$) for the second step of the sequential hydrogenation reaction over the Ru/Pd/MCMOS cascade catalysts with different Ru/Pd ratios. Reaction conditions: nitrobenzene (0.25 mmol), solid catalyst (Ru 0.77 mol%, Pd 1.01 mol%), ethanol (2.0 mL), H_2 (2.0 MPa), 80 ^{o}C.

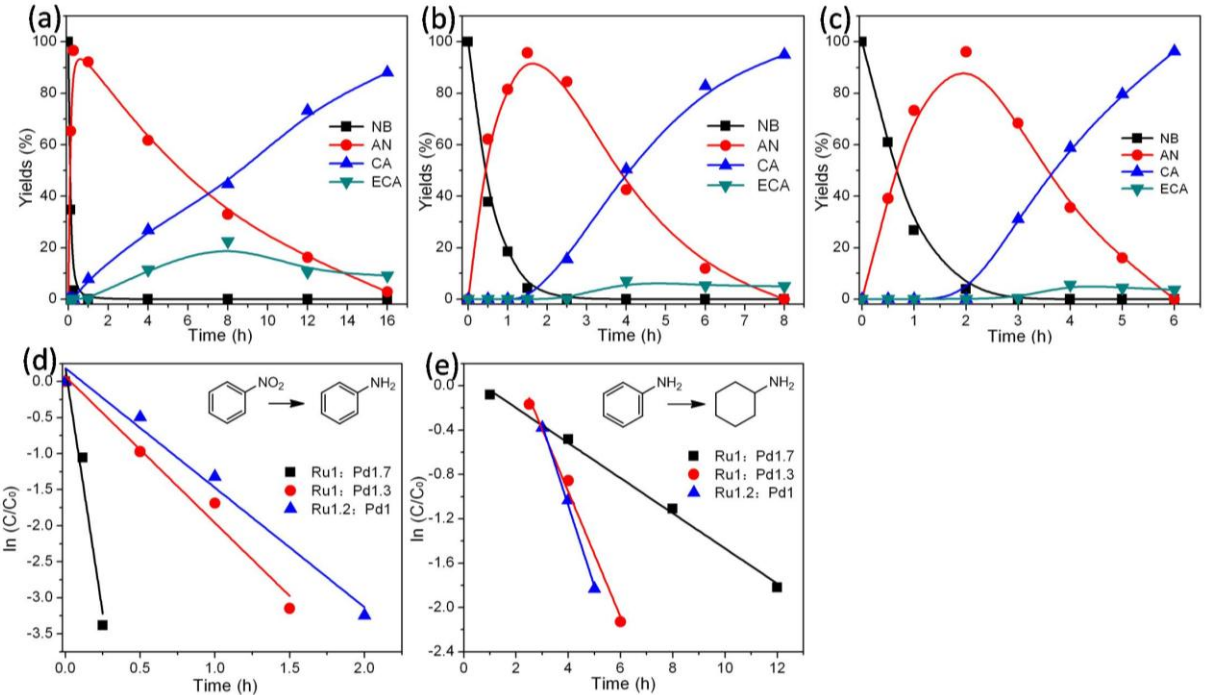

Supplementary Figure 35 | Catalytic performances evaluation. (a-c) Kinetic plots of the sequential hydrogenation of nitrobenzene to cyclohexylamine over the physically mixed catalyst with different Ru/Pd ratios: (a) Ru_1/MCMOS+$Pd_{1.7}$/MCMOS, (b) Ru_1/MCMOS+$Pd_{1.3}$/MCMOS and (c) $Ru_{1.2}$/MCMOS+Pd_1/MCMOS. (d) Plot of time vs ln($C/C_{0,\ NB}$) for the first step of the sequential hydrogenation reaction over the physically mixed catalyst with different Ru/Pd ratios. (e) Plot of time vs ln($C/C_{0,\ AN}$) for the second step of the sequential hydrogenation reaction over the physically mixed catalyst with different Ru/Pd ratios. Reaction conditions: nitrobenzene (0.25 mmol), solid catalyst (Ru 0.77 mol%, Pd 1.01 mol%), ethanol (2.0 mL), H_2 (2.0 MPa), 80 ^{o}C.

Supplementary Table 1 | Results of the sequential hydrogenation of nitrobenzene catalyzed by different catalysts.[a]

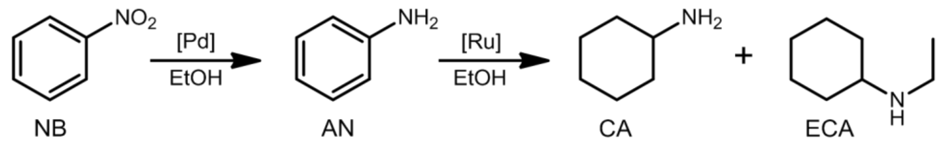

Entry	Catalyst	Conversion (%)	Yield (%)				CA production rate (h^{-1}) [b]
			AN	CA	ECA	Others	
1	Pd/MCMOS	11.2	11.2	trace	trace	trace	-
2	Ru/MCMOS	99.9	98.2	trace	trace	1.7	-
3	Ru/MCMOS + Pd/MCMOS[c]	99.9	84.5	15.5	trace	trace	3.6
4	Ru-Pd/DSNs[d]	99.9	61.2	34.0	4.8	trace	7.6
5	PdRu/MCMOS[e]	99.5	91.3	trace	trace	8.2	-
6	Ru/Pd/MCMOS	99.9	0.3	99.2	0.5	trace	26.5

[a] Reaction conditions: nitrobenzene (0.25 mmol), solid catalyst (Ru 0.77 mol%, Pd 1.01 mol%), ethanol (2.0 mL), H_2 (2.0 MPa), 80 ^{o}C, 2.5 h.

[b] The CA production rate is calculated according to the CA yield of 20-30%.

[c] Physically mixed catalyst.

[d] Pd NPs and Ru NPs are jointly loaded inside the channels of DSNs without spatial isolation.

[e] PdRu solid solution alloy NPs immobilized throughout the MCMOS.

Supplementary Methods

1. Chemicals

Tetraethylorthosilicate (TEOS, 98%), aqueous ammonia (NH_3 H_2O, 28%), cetyltrimethylammonium bromide (CTAB, 99.0 %), sodium borohydride ($NaBH_4$, 99%) and Poly(vinyl pyrrolidone) (PVP, MW=40000) were purchased from Sinopharm Chemical Reagent. Organosilanes including 1, 2-bis(triethoxysilyl)-ethane (BTEE, 96%), bis(triethoxysilyl)methane (BTEM, 96%), 1, 2-bis(triethoxysilyl)ethylene (BTEEE, 95%) and 3-aminopropyltriethyoxylsilane (APTES, 96%) was purchased from Gelest. Sodium tetrachloropalladate(II) (Na_2PdCl_4, 98%) and ruthenium(III) chloride hydrate ($RuCl_3$ H_2O, Ru≤37%) were purchased from Energy. All nitroaromatics were purchased from Aladdin. All chemicals were used as received without any further purification.

2. Material Synthesis

Synthesis of the Ru-Pd/DSNs catalyst with Ru and Pd NPs jointly loaded inside the channels of DSNs. The Ru-Pd/DSNs catalyst was prepared via a similar process for Ru/Pd/MCMOS. Firstly, the Ru/DSNs catalyst was prepared via a impregnation-reduction method, which is the same with the first step for preparing Ru/Pd/MCMOS. Then 4-8 nm of Pd nanoparticles were loaded into the Ru/DSNs catalyst via a simple adsorption process, which is the same with the third step for Ru/Pd/MCMOS. Typically, Pd nanoparticles solution obtained according to a previous report[8] was dropwise added into a solution containing 20 mL of H_2O and a given amount of Ru/DSNs. After stirring for 12 h at room temparature, the Ru-Pd/DSNs catalyst was collected by centrifugation and then washed with water and ethanol several times. After drying at 80 ^{o}C for 6 h under vacuum, the Ru-Pd/DSNs catalyst was obtained.

Synthesis of the PdRu/MCMOS alloy catalyst with PdRu alloy nanoparticles loaded in the MCMOS. The PdRu alloy nanoparticles were prepared via a representative publication[9]. Typically, 222 mg of PVP was dissolved in 50 mL of TEG and the solution was heated to 200 ^{o}C in oil bath with magnetic stirring. Meanwhile, 0.5 mmol of Na_2PdCl_4 and 0.5 mmol of $RuCl_3$ xH_2O were dissolved in 20 mL of water. Such a solution was then slowly added dropwise into the TEG (triethyleneglycol) at 200 ^{o}C in 40 min. After cooling to room temperature, acetone and alcohol were added into the TEG solution to precipitate the nanoparticles, followed by centrifugation. The

precipitation and centrifugation was repeated 5 times to remove unbound PVP. Finnaly, the obtained PdRu alloy nanoparticles were loaded in the MCMOS material via a same adsorption process for Ru/Pd/MCMOS and Ru-Pd/DSNs. After drying at 80 ^{o}C for 6 h under vacuum, the PdRu/MCMOS alloy catalyst was obtained.

Synthesis of RuO_x/MCMOS, RuO_x/DSNs and RuO_x/Pd/MCMOS. The RuO_x/MCMOS sample was prepared by partly oxidizing the Ru NPs of Ru/MCMOS using hypochlorous acid (HClO) according to a previous publication[7]. Typically, 200 mg of Ru/MCMOS were dispersed in 50 mL of water by ultrasonication. After the addition of 4 mL of a HClO solution, the resultant mixture was further stirred at room temperature for 24 h. The resultant RuO_x/MCMOS were collected through centrifugation and then washed with water several times. By a same method, the RuO_x/DSNs sample was obtained by partly oxidizing the Ru NPs of Ru/DSNs. The RuO_x/Pd/MCMOS sample was prepared by introducing Pd NPs in the RuO_x/MCMOS sample according to the method for Ru/Pd/MCMOS and Ru-Pd/DSNs. In these three samples, the RuO_x species and the Pd NPs were positioned in the nanocavities and the surface grooves of MCMOS, respectively.

3. Catalytic Tests of Both of Catalytic Steps

Selective hydrogenation of nitrobenzene to aniline. In a typical hydrogenation reaction, nitrobenzene (0.25 mmol), catalyst (12 mg, Pd 0.34 mol%) and ethanol (2.0 mL) was added to a Teflon-lined steel autoclave. Before each run, the autoclave was sealed and flushed with H_2 three times to remove the air. Then the autoclave was charged with a 2.0 MPa H_2 at room temperature, and then heated from room temperature to 80 ^{o}C within 20 min and kept at 80 ^{o}C under magnetic stirring (900 rpm). After reaction the autoclave was cooled down to room temperature, the products were analyzed by a gas chromatograph (Agilent 7890A) equipped with an HP-5 column, and further confirmed by GC-MS (Agilent 7890A GC/5973 MS).

Selective hydrogenation of aniline to cyclohexylamine. In a typical hydrogenation reaction, aniline (0.25 mmol), catalyst (12 mg, Ru 0.26 mol%) and ethanol (2.0 mL) was added to a Teflon-lined steel autoclave. Before each run, the autoclave was sealed and flushed with H_2 three times to remove the air. Then the autoclave was charged with a 2.0 MPa H_2 at room temperature, and then heated from room temperature to 80 ^{o}C within 20 min and kept at 80 ^{o}C under magnetic stirring (900 rpm). After

reaction the autoclave was cooled down to room temperature, the products were analyzed by a gas chromatograph (Agilent 7890A) equipped with an HP-5 column, and further confirmed by GC-MS (Agilent 7890A GC/5973 MS).

4. Materials Characterization

Scanning electron microscope (SEM) images were obtained on a JEOL JSM-6700F field-emission electron microscope. Transmission electron microscopy (TEM) images, high-angle annular dark-field scanning transmission electron microscopy (HAADF-STEM) images, and energy-dispersive X-ray spectroscopy (EDX) analyses were collected on an FEI Tecnai G^2 F20s-twin D573 field emission transmission electron microscope with an accelerating voltage of 200 kV. N_2 adsorption-desorption isotherms were obtained at -196 ^oC on a Micromeritics ASAP 2010 sorptometer. Samples were degassed at 120 ^oC for 12 h prior to analysis. Brunauer–Emmett–Teller (BET) surface areas were calculated from the linear part of the BET plot. ^{29}Si CP-MAS NMR measurements were performed on a Bruker AVANCE III 400 WB spectrometer. The spinning rate was 12 kHz and a total number of 20 000 scans were recorded with 6 s recycle delay for each sample. ^{13}C-MAS NMR measurements were performed on Varian Infinity Plus 400 NMR spectrometer. The spinning rate was 4 kHz and a total number of 800 scans were recorded with 4 s recycle delay for each sample. The FT-IR spectra were acquired using a Bruker IFS 66 V/SFTIR spectrometer in the range 400-4000 cm^{-1}. The thermogravimetric analysis (TGA) curve was obtained in a flow of air from 30 to 800 ^oC (10 ^oC min^{-1}) using a Netzsch STA 449F3 thermogravimetric analyzer. Inductively coupled plasma mass spectrometry (ICP-MS) analyses were carried out on a NexION 350 ICP-MS instrument. XPS analysis was carried out on an ESCALAB 250 X-ray photoelectron spectrometer with Al Kα as the excitation source. Temperature-programmed hydrogen reduction (H_2-TPR) and desorption (H_2-TPD) were conducted on a BETCAT-B adsorption apparatus. In H_2-TPR, the samples (50 mg) were heated from 30 to 300 ℃ with a heating rate of 10 ℃/ min under 30 mL/min of flowing 5% H_2/N_2. In H_2-TPD, the samples were pretreated at 350 ℃ under 5% H_2/N_2 for 2 h, purged with N_2 for 0.5 h, and then cooled down to -50 ℃ to adsorb H_2. After they were purged with N_2 for 30 min, the samples were heated to 200 ℃ at a rate of 10 ℃/min.

5. DFT Calculations

All calculations were carried out by using the projector augmented wave method in the framework of

the density functional theory (DFT),[10] as implemented in the Vienna *ab*-initio Simulation Package (VASP). The generalized gradient approximation (GGA) and Perdew–Burke–Ernzerhof (PBE) exchange functional was used. The plane-wave energy cutoff was set to 500 eV, and the Monkhorst–Pack method was employed for the Brillouin zone sampling. The convergence criterions of energy and force calculations were set to 10^{-5} eV/atom and 0.01 eV $Å^{-1}$, respectively. To explore the interactions between hydrogen molecule or organic molecules (nitrobenzene and aniline) and metal surface (cluster), including Pd (111) surface and Ru_{22} cluster, the adsorption energies of hydrogen molecule or organic molecules on the metal surface (cluster) were calculated. And the bader charge of adsorbed hydrogen atoms is also presented. The Pd (111) surface was built by the 4×4 slab unit-cell with four atomic layers[11]. The Ru_{22} cluster was modeled by a triangular pyramidalstructure with 22 Ru atoms[12]. A vacuum region of 15 Å is applied to avoid interactions between the neighboring configurations. Here, the adsorption energies (E_{ad}) were calculated by the energy difference of the system after and before adsorption[13]:

E_{ad} = E(organic molecule-metal) – E(organic molecule) – E(metal), where E(organic molecule-metal), E(organic molecule), and E(metal) represent the DFT energies of the organic molecule adsorbed metal surface, the energy of an isolated organic molecule, and the energy of the clean metal surface or cluster.

Supplementary Mass Spectrometry Data

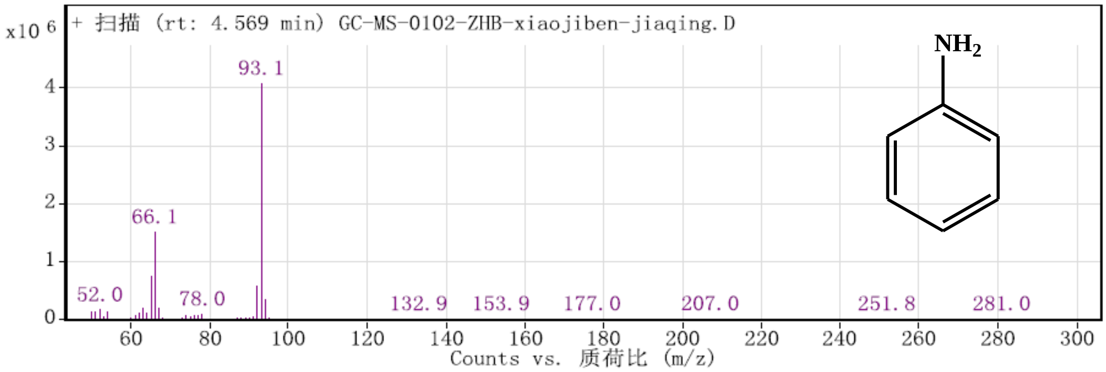

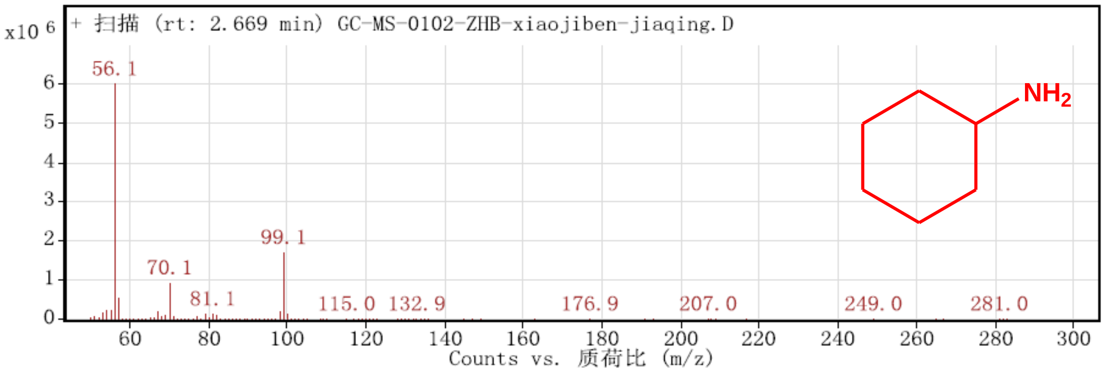

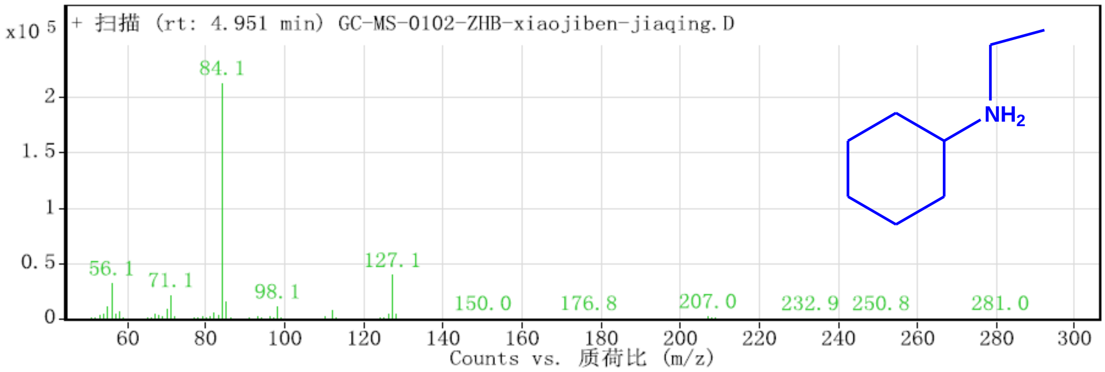

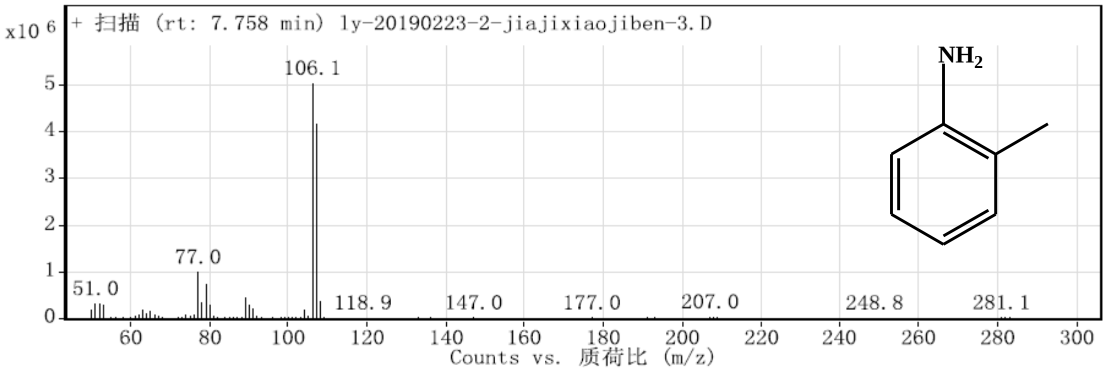
x10 6
+ 扫描 (rt: 7.758 min) ly-20190223-2-jiajixiaojiben-3.D
106.1
77.0
51.0
118.9
147.0
177.0
207.0
248.8
281.1
NH2
Counts vs. 质荷比 (m/z)

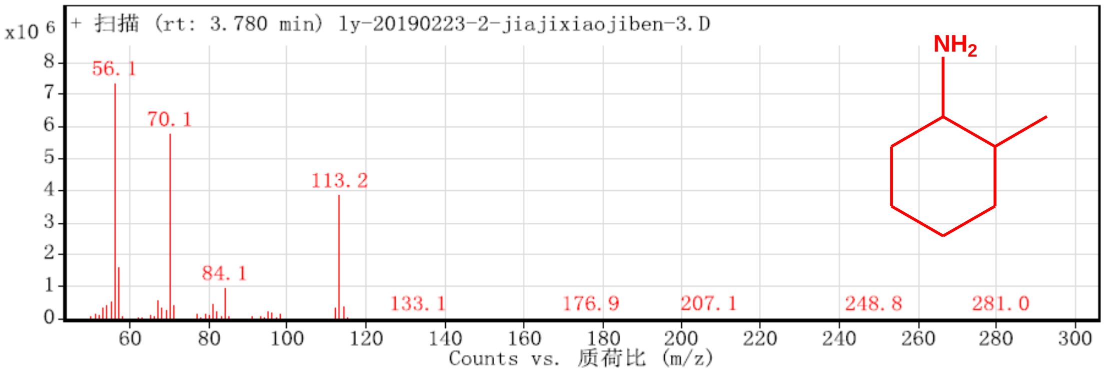
x10 6
+ 扫描 (rt: 3.780 min) ly-20190223-2-jiajixiaojiben-3.D
56.1
70.1
113.2
84.1
133.1
176.9
207.1
248.8
281.0
NH2
Counts vs. 质荷比 (m/z)

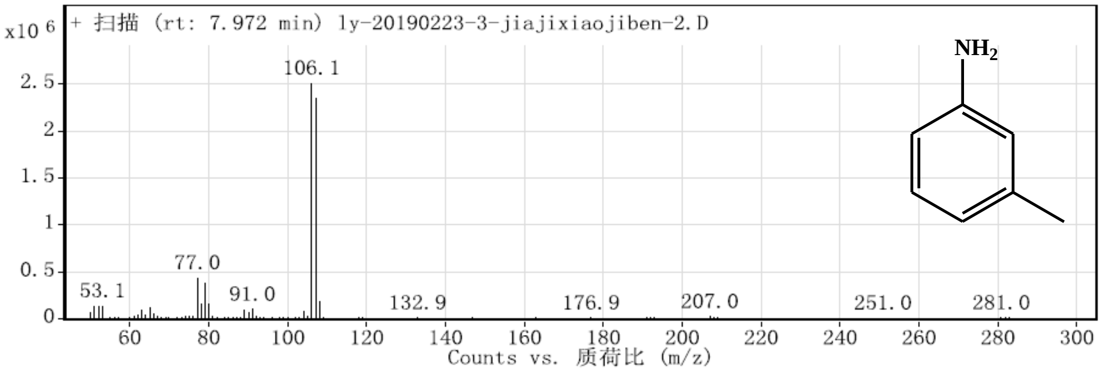
x10 6
+ 扫描 (rt: 7.972 min) ly-20190223-3-jiajixiaojiben-2.D
106.1
77.0
53.1
91.0
132.9
176.9
207.0
251.0
281.0
NH2
Counts vs. 质荷比 (m/z)

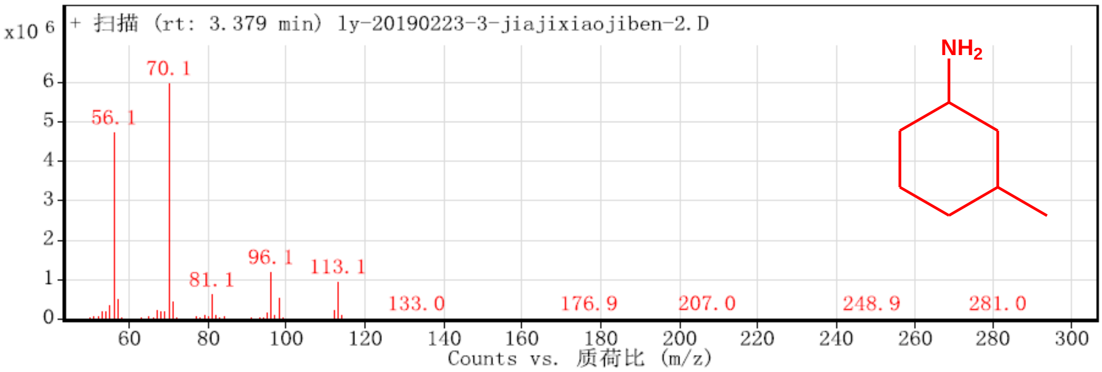
x10 6
+ 扫描 (rt: 3.379 min) ly-20190223-3-jiajixiaojiben-2.D
70.1
56.1
96.1
81.1
113.1
133.0
176.9
207.0
248.9
281.0
NH2
Counts vs. 质荷比 (m/z)

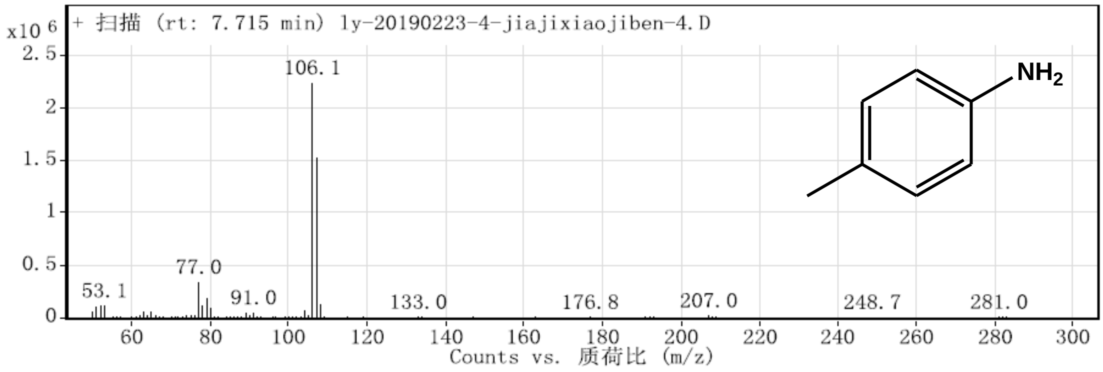
x10 6
+ 扫描 (rt: 7.715 min) ly-20190223-4-jiajixiaojiben-4.D
106.1
77.0
53.1
91.0
133.0
176.8
207.0
248.7
281.0
NH2
Counts vs. 质荷比 (m/z)

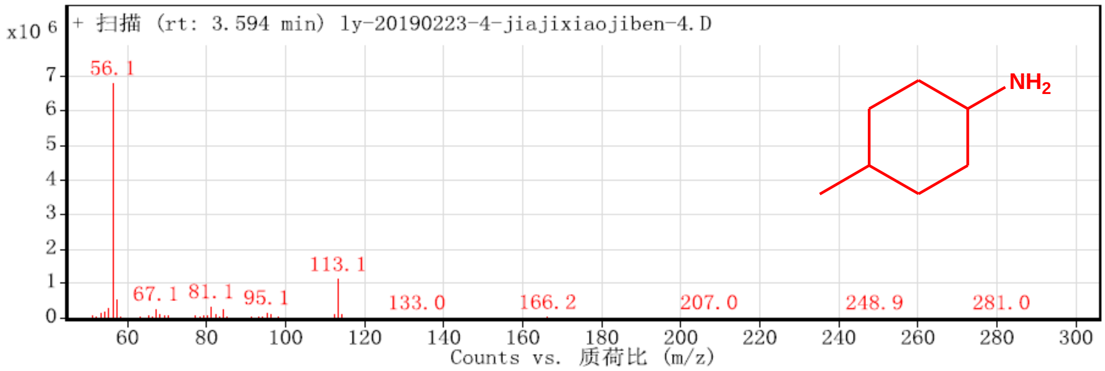
x10 6
+ 扫描 (rt: 3.594 min) ly-20190223-4-jiajixiaojiben-4.D
56.1
113.1
67.1
81.1
95.1
133.0
166.2
207.0
248.9
281.0
NH2
Counts vs. 质荷比 (m/z)

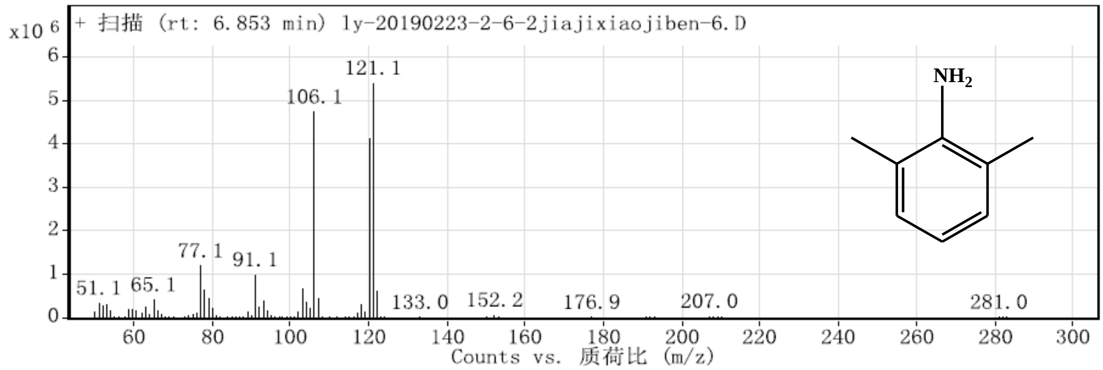
x10 6
+ 扫描 (rt: 6.853 min) ly-20190223-2-6-2jiajixiaojiben-6.D
121.1
106.1
77.1
91.1
51.1
65.1
133.0
152.2
176.9
207.0
281.0
NH2
Counts vs. 质荷比 (m/z)

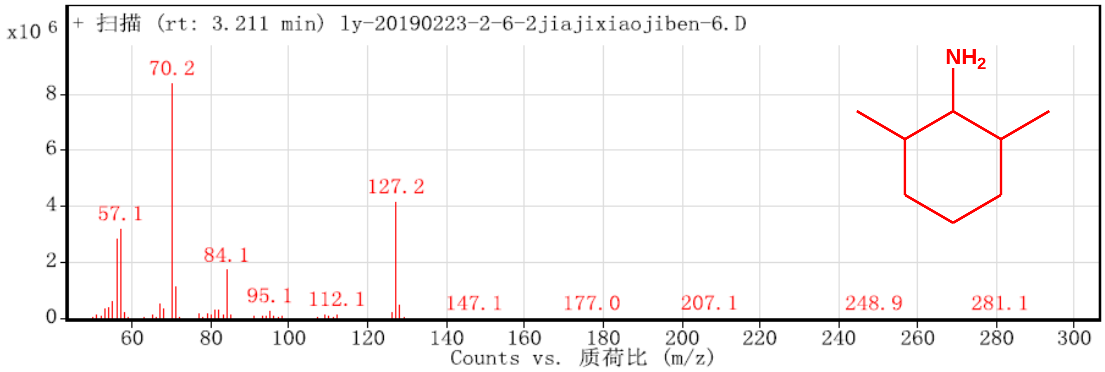
x10 6
+ 扫描 (rt: 3.211 min) ly-20190223-2-6-2jiajixiaojiben-6.D
70.2
127.2
57.1
84.1
95.1
112.1
147.1
177.0
207.1
248.9
281.1
NH2
Counts vs. 质荷比 (m/z)

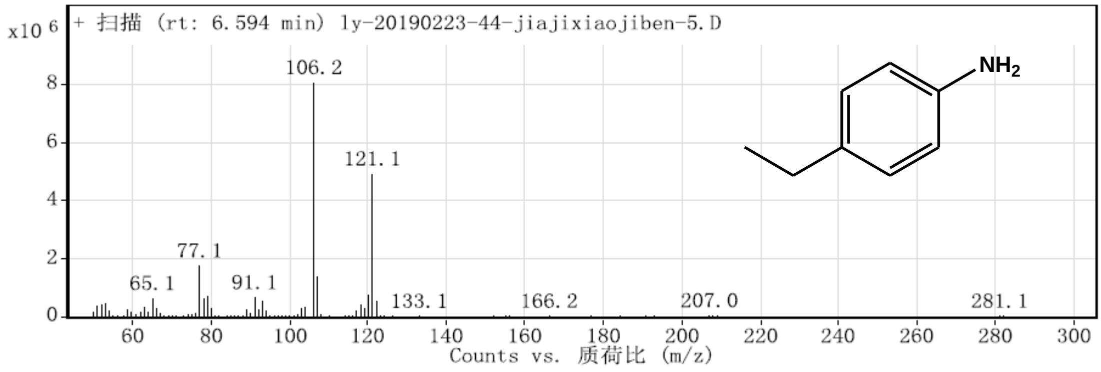

x10 6
+ 扫描 (rt: 6.594 min) ly-20190223-44-jiajixiaojiben-5.D
106.2
121.1
77.1
65.1
91.1
133.1
166.2
207.0
281.1
Counts vs. 质荷比 (m/z)
NH2

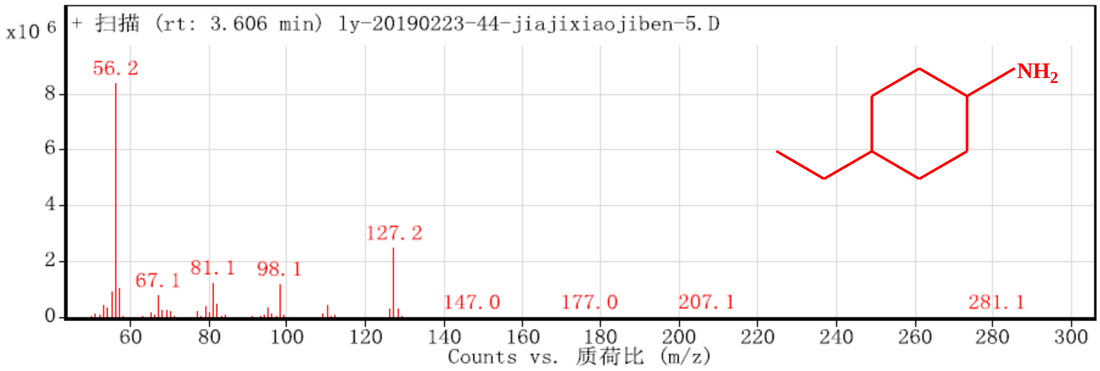

x10 6
+ 扫描 (rt: 3.606 min) ly-20190223-44-jiajixiaojiben-5.D
56.2
127.2
67.1
81.1
98.1
147.0
177.0
207.1
281.1
Counts vs. 质荷比 (m/z)
NH2

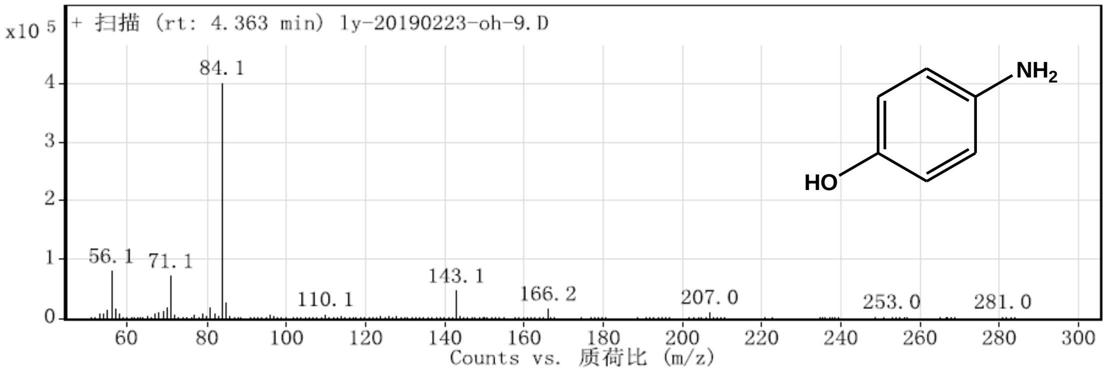

x10 5
+ 扫描 (rt: 4.363 min) ly-20190223-oh-9.D
84.1
56.1
71.1
110.1
143.1
166.2
207.0
253.0
281.0
Counts vs. 质荷比 (m/z)
NH2
HO

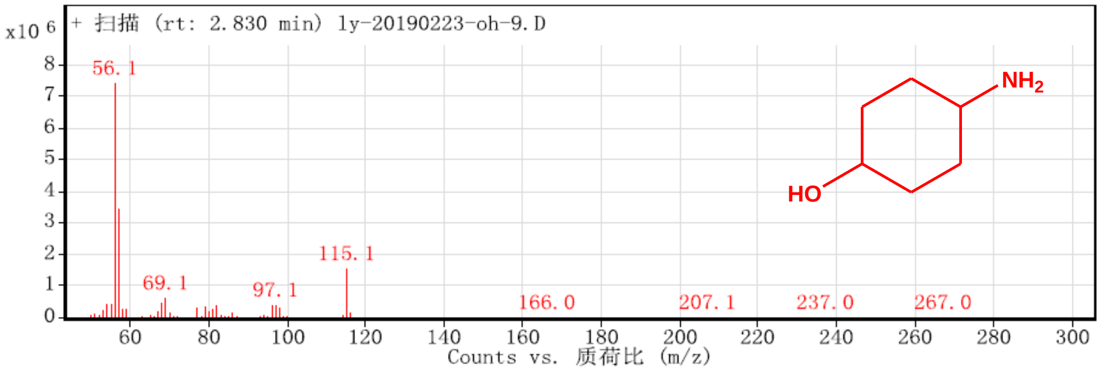

x10 6
+ 扫描 (rt: 2.830 min) ly-20190223-oh-9.D
56.1
69.1
97.1
115.1
166.0
207.1
237.0
267.0
Counts vs. 质荷比 (m/z)
NH2
HO

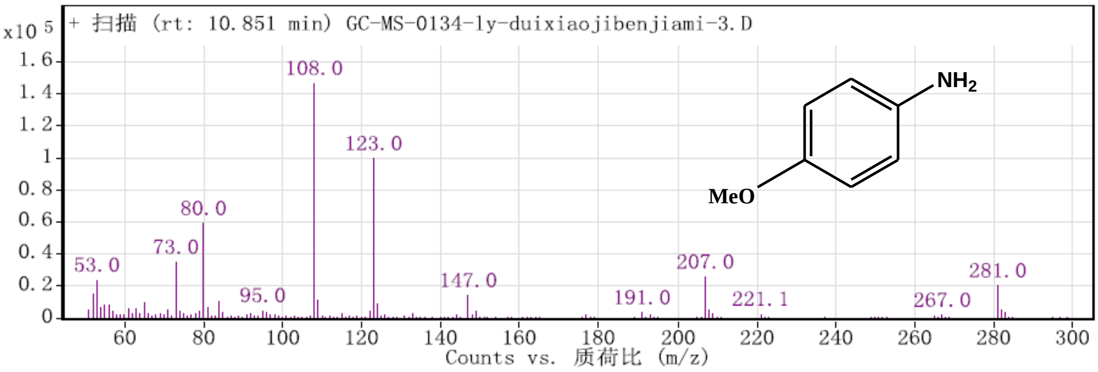
x10 5
+ 扫描 (rt: 10.851 min) GC-MS-0134-ly-duixiaojibenjiami-3.D
108.0
123.0
80.0
73.0
53.0
95.0
147.0
191.0
207.0
221.1
267.0
281.0
NH_2
MeO
Counts vs. 质荷比 (m/z)

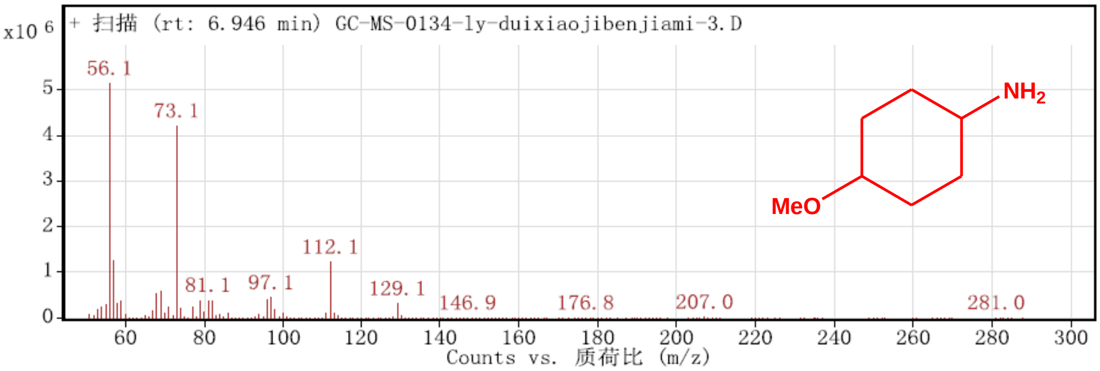
x10 6
+ 扫描 (rt: 6.946 min) GC-MS-0134-ly-duixiaojibenjiami-3.D
56.1
73.1
112.1
81.1
97.1
129.1
146.9
176.8
207.0
281.0
NH_2
MeO
Counts vs. 质荷比 (m/z)

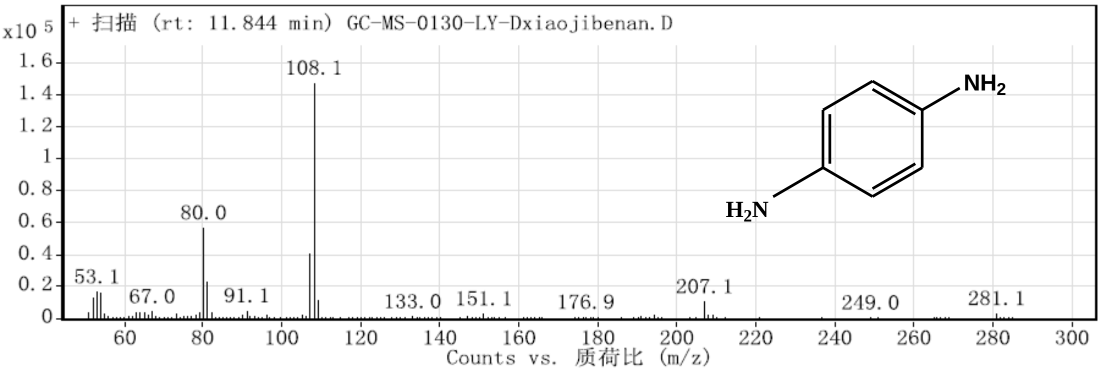
x10 5
+ 扫描 (rt: 11.844 min) GC-MS-0130-LY-Dxiaojibenan.D
108.1
80.0
53.1
67.0
91.1
133.0
151.1
176.9
207.1
249.0
281.1
NH_2
H_2N
Counts vs. 质荷比 (m/z)

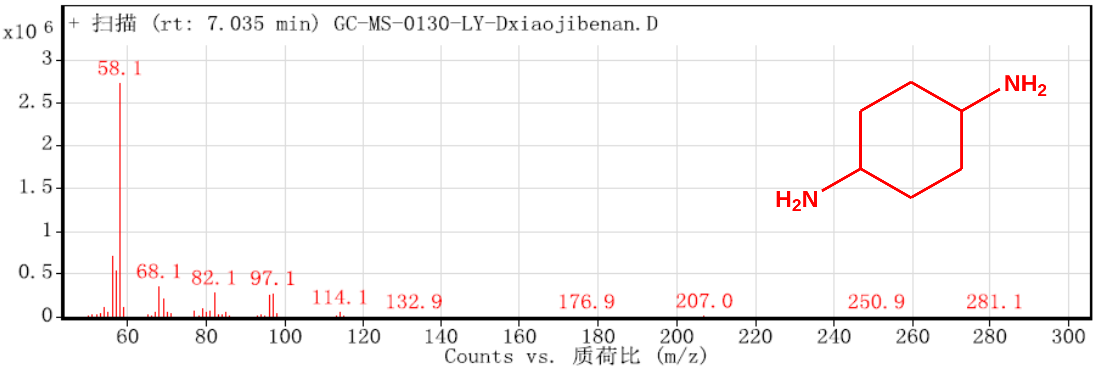
x10 6
+ 扫描 (rt: 7.035 min) GC-MS-0130-LY-Dxiaojibenan.D
58.1
68.1
82.1
97.1
114.1
132.9
176.9
207.0
250.9
281.1
NH_2
H_2N
Counts vs. 质荷比 (m/z)

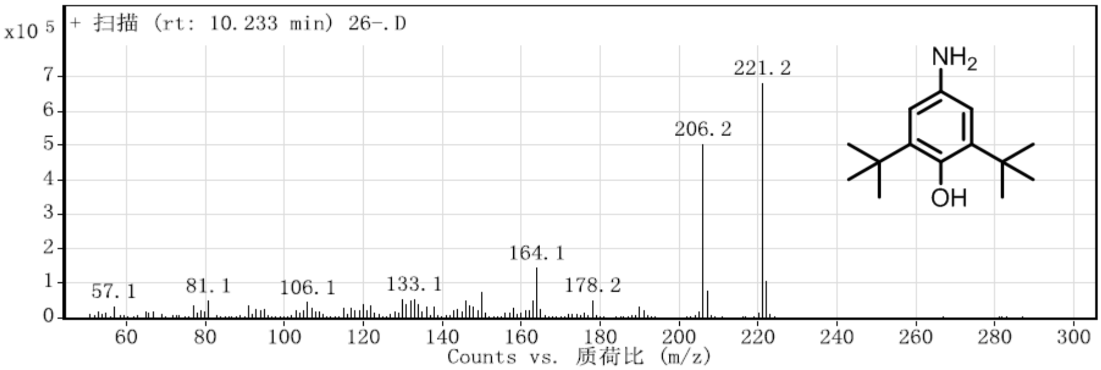
x10 5
+ 扫描 (rt: 10.233 min) 26-.D
221.2
206.2
164.1
57.1
81.1
106.1
133.1
178.2
NH2
OH
Counts vs. 质荷比 (m/z)

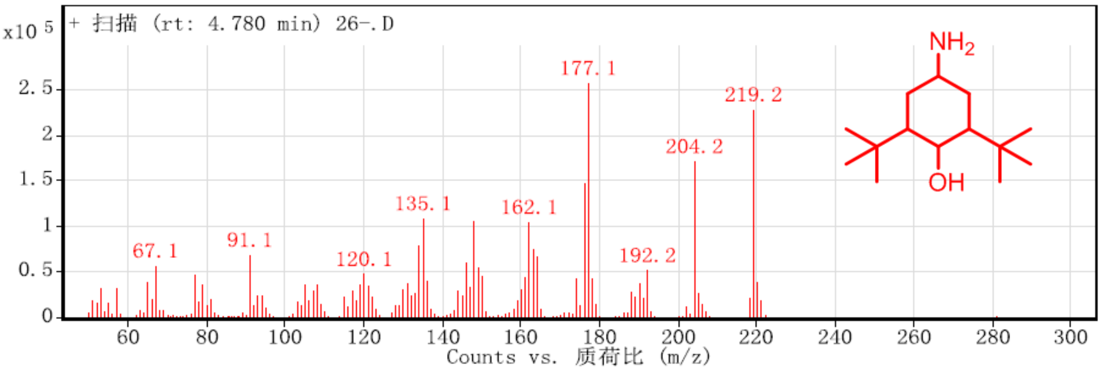
x10 5
+ 扫描 (rt: 4.780 min) 26-.D
177.1
219.2
204.2
135.1
162.1
67.1
91.1
120.1
192.2
NH2
OH
Counts vs. 质荷比 (m/z)

Supplementary References

1. Zou, H. et al. An organosilane-directed growth-induced etching strategy for preparing hollow/yolk-shell mesoporous organosilica nanospheres with perpendicular mesochannels and amphiphilic frameworks. *J. Mater. Chem. A.* **2**, 12403–12564 (2014).

2. Zou, H., Wang, R., Shi, Z., Dai, J., Zhang, Z. & Qiu, S. One–dimensional periodic mesoporous organosilica helical nanotubes with amphiphilic properties for the removal of contaminants from water. *J. Mater. Chem. A.* **4**, 4145–4154 (2016).

3. Yang, Y., Liu, J., Li, X. B., Liu, X. & Yang, Q. H. Organosilane-assisted transformation from core–shell to yolk–shell nanocomposites. *Chem. Mater.* **23**, 3676–3684 (2011).

4. Liu, J. et al. Yolk–shell hybrid materials with a periodic mesoporous organosilica shell: Ideal nanoreactors for selective alcohol oxidation. *Adv. Funct. Mater.* **22**, 591–599 (2012).

5. Komanoya, T., Kinemura, T., Kita, Y., Kamata, K. & Hara, M. Electronic effect of ruthenium nanoparticles on efficient reductive amination of carbonyl compounds. *J. Am. Chem. Soc.* **139**, 11493–11499 (2017).

6. Robinson, A., Mark, L., Rasmussen, M., Hensley, J. & Medlin, J. Surface chemistry of aromatic reactants on Pt- and Mo-modified Pt catalysts. *J. Phys. Chem. C* **120**, 26824−26833 (2016).

7. Bai, J.; Han, S.; Peng, R.; Zeng, J.; Jiang, J.; Chen, Y. Ultrathin rhodium oxide nanosheet nanoassemblies: Synthesis, morphological stability, and electrocatalytic application. *ACS Appl. Mater. Interfaces* **9**, 17195–17200 (2017).

8. Shao, M., Yu, T., Odell, J., Jin, M. & Xia, Y. Structural dependence of oxygen reduction reaction on palladium nanocrystals. *Chem. Commun.* **47**, 6566–6568 (2011).

9. Kusada, K. et al. Solid solution alloy nanoparticles of immiscible Pd and Ru elements neighboring on Rh: Changeover of the thermodynamic behavior for hydrogen storage and enhanced CO-oxidizing ability. *J. Am. Chem. Soc.* **136**, 1864–1871 (2014).

10. Kohn, W. & Sham, L. J. Self-consistent equations including exchange and correlation effects. *Phys. Rev.* **140** (4A), A1133−A1138 (1965).

11. Li, G., Han, J., Wang, H., Zhu, X. & Ge, Q. Role of dissociation of phenol in its selective hydrogenation on Pt(111) and Pd(111). *ACS Catal.* **5**, 2009−2016 (2015).

12. Zhang, S.-T., Li, C.-M., Yan, H., Wei, M., Evans, D. G. & Duan, X. Density functional theory study on the metal–support interaction between Ru cluster and anatase TiO_2 (101) surface. *J. Phys. Chem. C* **118**, 3514−3522

(2014).

13. Lv, X., Xu, Z., Li, J., Chen, J. & Liu, Q. Investigation of fluorine adsorption on nitrogen doped $MgAl_2O_4$ surface by first-principles. *Appl. Surf. Sci.* **376**, 97−104 (2016).
